# Seasonal microbial dynamics in the ocean inferred from assembled and unassembled data: a view on the unknown biosphere

**DOI:** 10.1038/s43705-022-00167-8

**Published:** 2022-09-21

**Authors:** Didier Debroas, Corentin Hochart, Pierre E. Galand

**Affiliations:** 1 Université Clermont Auvergne, CNRS, Laboratoire Microorganismes: Genome et Environnement, 63000 Clermont-Ferrand, France; 2 Sorbonne Universités, CNRS, Laboratoire d’Ecogéochimie des Environnements Benthiques (LECOB), Observatoire Océanologique de Banyuls, Banyuls sur Mer, France

**Keywords:** Metagenomics, Microbial ecology

## Abstract

In environmental metagenomic experiments, a very high proportion of the microbial sequencing data (> 70%) remains largely unexploited because rare and closely related genomes are missed in short-read assemblies. The identity and the potential metabolisms of a large fraction of natural microbial communities thus remain inaccessible to researchers. The purpose of this study was to explore the genomic content of unassembled metagenomic data and test their level of novelty. We used data from a three-year microbial metagenomic time series of the NW Mediterranean Sea, and conducted reference-free and database-guided analysis. The results revealed a significant genomic difference between the assembled and unassembled reads. The unassembled reads had a lower mean identity against public databases, and fewer metabolic pathways could be reconstructed. In addition, the unassembled fraction presented a clear temporal pattern, unlike the assembled ones, and a specific community composition that was similar to the rare communities defined by metabarcoding using the 16S rRNA gene. The rare gene pool was characterised by keystone bacterial taxa, and the presence of viruses, suggesting that viral lysis could maintain some taxa in a state of rarity. Our study demonstrates that unassembled metagenomic data can provide important information on the structure and functioning of microbial communities.

## Introduction

Metagenomics studies are based on gene centric approaches often based on assembly followed by contigs binning for building metagenome-assembled genomes (MAGs). However, a relatively low proportion of the reads can be assembled into contigs or/and MAGs. Often the higher proportion of the sequencing data (>70%) remains largely unexploited in metagenomes because rare and closely related genomes are missed in short-read data assemblies [1]. Indeed, a minimum sequencing depth is often needed for contig assembly. Bacterial species with coverage below 15x in metagenomes typically result in low-quality assemblies [2]. For Luo et al. [3], a species can only be accurately assembled from a complex metagenome when it shows at least 20x coverage. Since rare species within a community typically have low sequencing coverage, they are hardly assembled into long contigs. To reconstruct rare strains from complex assemblages thus requires sometimes an enormous dataset with a very high coverage depth exceeding sometime 1000x [4]. The approach described by Nielsen [5] allows, however, the reconstruction of any species with an adequate sequencing depth (~50x according to the simulation) and permits the binning of some rare members with the rarest having 0.02% relative abundance. However, a minimum sequencing depth is often needed, but not always sufficient for accurate contig assembly. Globally, assemblers perform poorly in the presence of multiple similar genomes from closely related species. In that case, unassembled reads can also belong to the flexible or accessory genome of the main components of the community. For instance, members of the wide spread marine *Prochlorococcus* genus have a huge pangenome, with ~1000 common genes (core genomes), and a ‘flexible’ genome, which is found in only one or a few of the *Prochlorococcus* genomes [6]. However, by comparing long and short reads, Sharon et al. [1] concluded that the majority of unassembled reads in the short-read data were left unassembled because of low coverage and not because of the presence of multiple similar regions.

The rare components of the metagenomics data, bacterial taxa (i.e. rare biosphere) or individual genes (i.e. flexible genome), which may be hard to assemble, could nevertheless play an important role in ecosystem functioning. Regarding genes for instance, genomic and metagenomic data have defined at least 12 major clades among *Prochlorococcus* and the flexible gene distribution within these clades determines adaptation to the local environment (light, temperature…) [6]. Genes present in the flexible gene pool, which are not abundant, are still important because they are often associated with specific nutritional requirements (phosphorus, nitrogen or iron, [6]). At the taxa level, rare populations of microorganisms, with their tremendous diversity [7], can also play an important role in ecosystem functioning. The “rare microbial biosphere” [8] was first seen mainly as a seed bank in which some members became dominant at times depending on specific environmental factors [9]. Some bacteria, for instance, become dominant under anthropogenic pressure [10] or when colonizing a new substrate [11]. Other changes in abundance can occur following climatic fluctuations [12]. These observations illustrate a transient state of rare microorganisms toward the abundant biosphere, or an oscillation within a rare state [13]. Inversely, some rare taxa always remain rare [13]. The fact that some of them exhibit high cell-level metabolic activity [14] could indicate that they are keystone species in ecosystems. Keystone taxa are defined by Banerjee et al. [15] as highly connected taxa that exert a considerable influence on microbiome structure and function, irrespective of their abundance across space and time. Thus, some low-abundance taxa that are highly connected in microbial communities can explain compositional turnover better than all the taxa combined [16]. However, the functional role of rare microorganisms remains poorly understood, since they are often phylogenetically distant from referenced cultured or uncultured microbes [14, 17, 18]. Therefore, the microbial rare biosphere may constitute an important genomic reservoir or diversity pool, and a source of genetic novelty with biotechnological potential [19, 20]. Thus, the rare taxa are certainly an important component of the “dark matter” [21], but the metabolic potential of the rare biosphere remains under-explored. A limited number of studies have focused on the genetic content of this biosphere [22, 23].

In this work, we focused on the rare genetic material defined here as the sequencing reads that do not align with assembled contigs. We hypothesize that this genetic material plays an important role in the marine ecosystem functioning. For this purpose, we analyzed a three-year metagenomic time series based on monthly samples from the Bay of Banyuls sur Mer (NW Mediterranean Sea).

## Materials and methods

### Sampling and sequencing

The sampling strategy was described in Galand et al. [24]. Briefly, surface seawater (3 m) was collected monthly from January 2012 to February 2015 (40 samples) by using a 10-L Niskin bottle at the SOLA station (42°31′N, 03°11′E) in the Bay of Banyuls sur Mer (France) in the northwestern Mediterranean. A volume of 5 L was prefiltered through 3-μm pore-size polycarbonate filters (Millipore, Billerica, MA, USA), and the microbial biomass was collected on 0.22-μm pore-size GV Sterivex cartridges (Millipore) and stored at −80 °C until nucleic acid extraction. The physicochemical parameters (Table S1) were provided by the “Service d’Observation en Milieu Littoral” (SOMLIT). After DNA extraction [24] samples were sequenced on eight lanes of a HiSeq 2500 “High-Output” paired-end run (2 × 100 bp). Raw reads were archived in the ENA repository under accession number PRJEB26919.

### Assembling

**Fig. 1:**
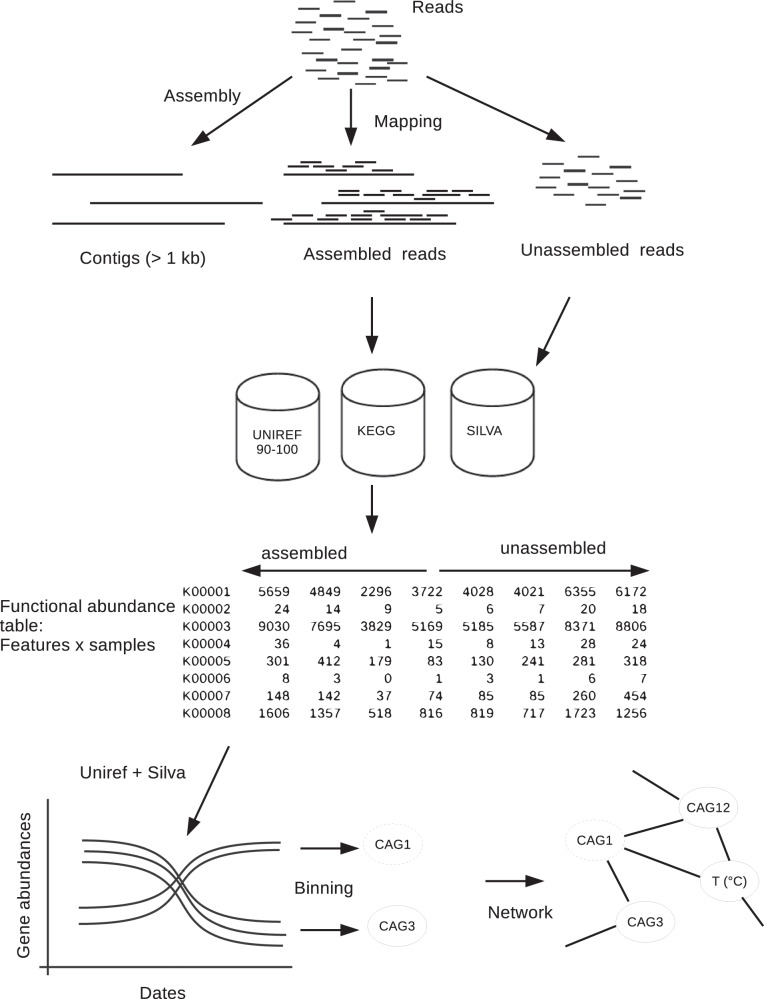
Bioinformatics pipeline. Schematic showing the bioinformatic analysis conducted to separate assembled and unassembled reads from a 3-year metagenomic time series dataset. The reads were mapped against contigs and functional and OTUs tables were built with assembled and unassembled reads. From these tables Co-Abundance gene Groups (CAGs) were inferred.

Raw paired-end Illumina reads were preprocessed by removing Nextera adapters with the bbduck program from the BBTools package (12.10.2015 release) (http://jgi.doe.gov/data-and-tools/bbtools/). Reads were then trimmed and filtered using Trimmomatic v. 0.33 [25] based on their quality generating a read length of ca. 85 bp. A total of 34 to 112 million reads per sample remained after filtering (Table S2). For each metagenome, high-quality reads were assembled into contigs with IDBA-UD [26] with the default iterative k-mer assembly and k-mer length increasing from 20 to 100 in steps of 20, the correction option, and with both pair-end reads (-r entry) and single-end reads (--long entry). Two kinds of reads were discriminated by mapping all the reads against the built contigs (Fig. 1). The mapping was conducted with bwa mem algorithm [27] with default parameters, the results by sample are displayed in Table S2. Thereafter, we term the two fractions as unassembled, as the pool of reads that do not match with contigs formed post-assembly, and assembled reads. However, algorithms implemented in mappers are different from assemblers and in some cases it can exist some discrepancies between these tools.

### Community composition, functional abundance table and OTU abundance table inferred from assembled and unassembled reads

The composition of the unassembled and assembled read fractions were compared to each other with MetaFast [28], which allows a direct reference-free comparison of shotgun metagenomic data. The Bray-Curtis dissimilarity matrix computed by MetaFast was used to construct a non-metric multidimensional scaling (NMDS) ordination with the vegan package in R [29].

An OTU abundance table based on 16 S rRNA gene were built for assembled and non assembled reads separately. The 16 S rRNA gene were identified by comparing all preprocessed reads to the SILVA database [30] with BLASTn (identity ≥ 90% and length > 80 bp). An abundance table was built by clustering reads at a 97% similarity against the SILVA sequence collection. In addition, a phylogenetic analysis was conducted based on unique clade-specific marker genes for assembled and unassembled reads with metaphlan2 [31], and the list of taxa and their relative abundance was used with LefSe [32] to identify the taxa that best explained the differences between the fractions. A functional abundance table was built with a reference-guided approach based on the UNIREF (90 and 100) [33] and KEGG databases [34]. Reads were compared against the databases using DIAMOND [35] with the blastx mode and the following parameters: -evalue 1e-5 --sensitive --max-target-seqs 1. Each function in these tables contains reads originating from multiple genomes. The generated abundance tables were characterized by zero-inflation. We removed all genes present as singletons only in the 80 samples (40 assembled and 40 unassembled), or detected in less than 20 samples. Gene loss are presented in Table S3. Overall, we counted 846 16 S rRNA OTUs, 6984 KOs, and 1,210,645 proteins (UNIREF90) in the entire dataset after applying strict filters described in the experimental procedures section (Table S3). The statistical analysis was conducted with the ALDEx2 methods [36] that take into account the compositional nature of the data [37]. Differences in abundance between the two categories of genes (derived from assembled and unassembled reads) were considered as significant (P < 0.05) when the Welch and Wilcoxon tests were convergent. The significant results annotated against the KEGG database were used to discriminate metabolic pathways between assembled and unassembled fractions with the “gage” and “pathview” functions implemented in R [38, 39].

Multivariate analyses were conducted with the R MixOmics package [40] by using the “spca” function with centered log ratio transformation (CLR) after replacing zeros with the “cmultRepl” function and the “czm” option included in the zCompositions library [41].

### Binning covarying gene groups with assembled and unassembled reads

The most common approach to reconstruct genomes from metagenomes is to build MAGs. MAG construction is based on mapping reads to contigs, but since we cannot obtain contigs from the rare reads, we chose an alternative approach to survey the potential genomic content of the communities. Co-Abundance gene Groups (CAGs) were built separately for the assembled and non assembled datasets, from the table gathering the functional abundance (UNIREF90) and OTU (SILVA) tables, with 3 different approaches: MSPminer [42], canopy [5] and Partial Least Squares regression (PLS) based networks. MSPminer and canopy bin covarying genes by a robust measure of proportionality or correlation between genes, and give a same weight to the proteins and rRNA genes. In our approach, unlike in the original methods cited, we used the abundance of functions rather than a gene catalog. In addition, we introduce a new method to bin genes from abundance tables by associating a Partial Least Squares regression (PLS) and a bipartite network. PLS relates the OTUs (16 S rRNA) and the protein tables. The goal was to predict the protein variations from the OTUs dynamics. The regression was computed with the “spls” function associated to the regression method in the MixOmics package in R [40]. In a second step, a bipartite network based on PLS was built linking OTUs and protein genes. The edges with a weight lower than 0.8 and orphan vertices were deleted by using the igraph package [43]. A CAG was then defined by grouping all the protein genes associated to one OTU.

The quality (completeness and contamination) of the CAGs built by these 3 different approaches were checked with checkM [44] with the option “--genes”. In a first step, 149 CAGs were defined and the taxonomy, completeness and contamination was assessed by checkM (Table S4). The temporal dynamics of these different CAGs were assessed from the median of the gene counts at each sampling date, and a network was built based on Spearman correlations. CAGs were considered redundant if their weight (i.e. correlation) in the network was higher than 0.95 to a CAG with the same taxonomy and amino acids identity >95%. This identity was computed with compareM (https://github.com/dparks1134/CompareM). These criteria were based on the histogram of the edge weight (i.e. correlations), manual inspection of the network cluster for the CAG taxonomy and the amino acid identity. The final network, with a correlation coefficient >0.8 or <−0.8 between edges, included 114 CAGs as well as 3 physicochemical parameters of the water samples. The centrality indices were computed with the package qgraph [45].

### Amplicon sequencing

Amplicon sequencing data were originally published in Lambert et al. [46]. Briefly, specific primer pairs 27F (5ʹ-AGRGTTYGATYMTGGCTCAG) and 519R (5ʹ-GTNTTACNGCGGCKGCTG) were used to target the V1-V3 regions of the bacterial 16S rRNA gene and sequencing was carried out with Illumina MiSeq 2 × 300 bp kits. The analysis of the raw reads was done by constructing amplicon sequence variants (ASVs) following the standard pipeline of the DADA2 package [47]. Abundant ASVs were defined as the ones with a representation >0.01% within a sample, and rare ASVs as having an abundance <0.01% within a sample [48].

## Results

### Temporal dynamics of the assembled and unassembled reads

**Fig. 2:**
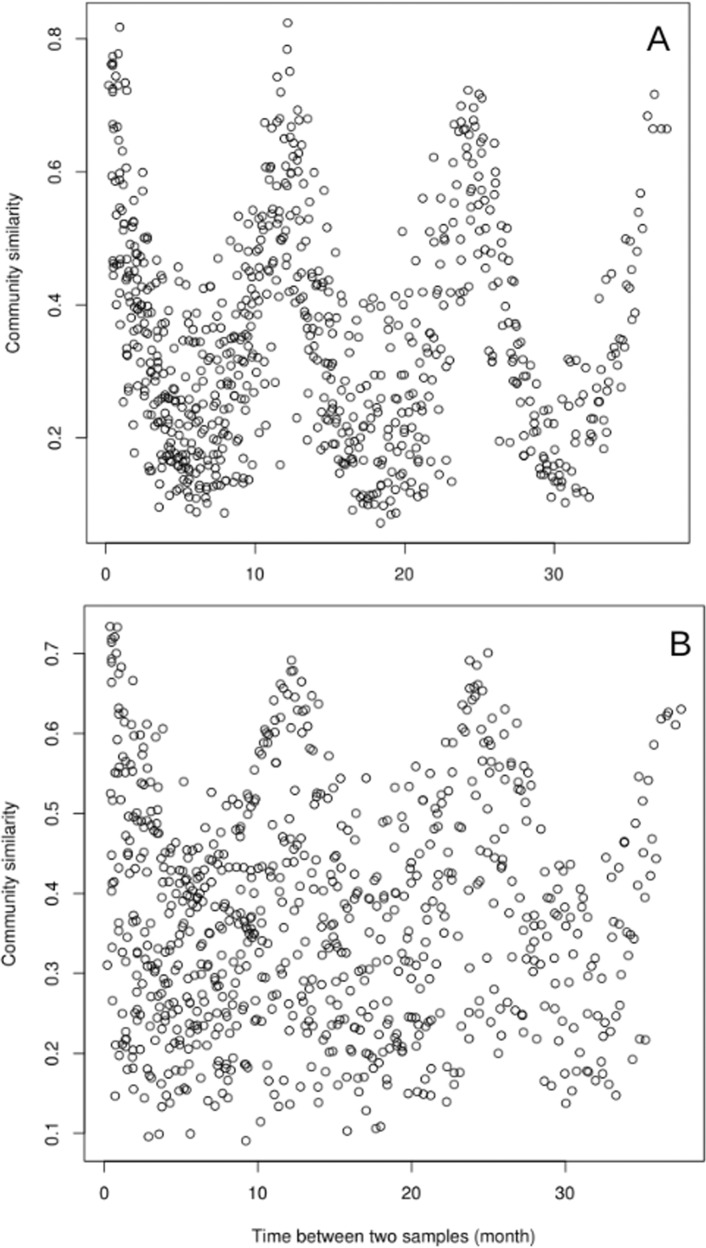
Pairwise comparisons of similarity between communities in relation to the time separating two samples. The similarity was measured by a direct metagenome-to-metagenome comparison of the read content for the unassembled (**A**) and assembled ones (**B**).

The reads from the three-year metagenomic time series were classified according to their mapping or not to contigs larger than 1 kb (i.e. assembled and unassembled) (Fig. 1). A direct comparison of the read composition between time points showed that for the unassembled reads the similarity between samples was highest when samples were taken one year apart (Fig. 2), and similarity was lowest when samples were taken six months apart (Fig. 2A). For the assembled reads, the seasonal pattern of similarity was noisy and the overall pattern was not as clear (Fig. 2B).

The non-metric multidimensional scaling (NMDS) computed from Bray-Curtis index obtained with MetaFast showed that the read composition of the unassembled fraction was different from the read composition of the assembled fraction (Fig. S1). We then identified the reads that were significantly enriched in each fraction (Table 1). From the statistical analysis (ALDEx2 methods) we deduced that a total of 130,450 proteins (10.7% of the total) were significantly enriched in the unassembled fraction and 125,953 (10.4%) in the assembled fraction. Furthermore, 26 16 S rRNA (mean reads: 170.5) and 25 KEGG (mean reads: 69.8) annotated genes were only present in the unassembled fraction. Conversely, 2523 UNIREF genes (mean: 209.2) were present only in the assembled fraction (Table 1).

**Table 1:** Distribution of the SILVA, UNIREF90 and KEGG clusters among the mapped and unmapped reads.

	Number of features	Only in the unassembled fraction	Only in the assembled fraction	Significant features	Enriched in the unassembled fraction	Enriched in the assembled fraction
SILVA (16S)	846	26	0	253	142	111
KEGG	6984	25	0	1944	516	1428
UNIREF90	1,210,645	7793	2523	256,403	130,450	125,953

Differences between both categories were considered significant (*P* < 0.05) when the Welch and Wilcoxon tests were convergent; the enrichment were inferred from the log fold computed by the ALDEx2 procedure.

### Taxonomic composition

**Fig. 3:**
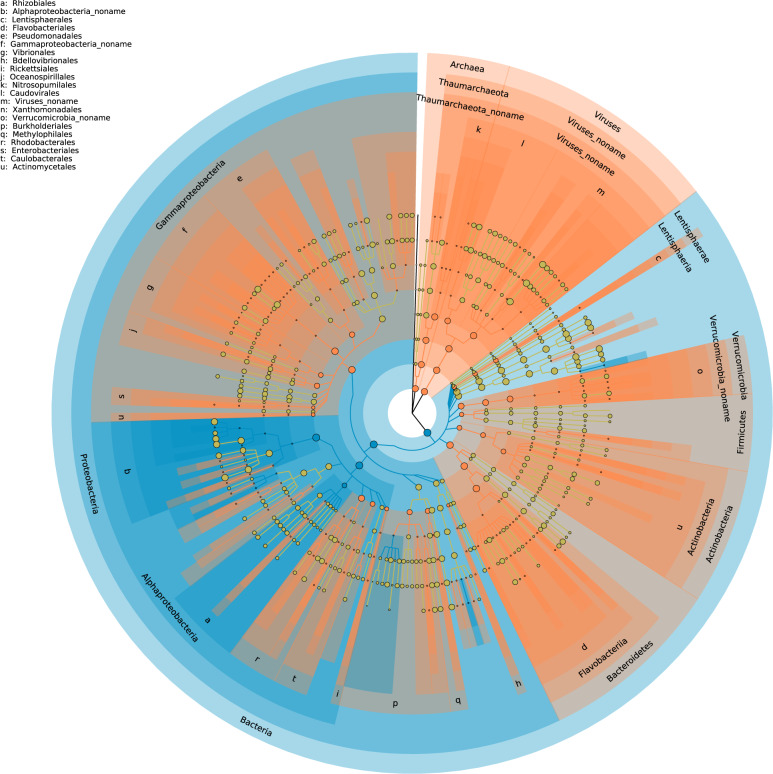
Cladogram showing the taxonomic position of the unassembled (orange) and assembled (blue) fractions and their relative abundance. Each circle diameter is proportional to the taxon’s abundance, and the color represents which branch of the phylogenetic tree is more abundant in each fraction.

To study the taxonomic composition of the two fractions, we used statistical analysis based on both unique clade-specific marker genes (Fig. 3) and rRNA genes (Fig. S2) found in the reads. In addition, we analyzed the results obtained from high-throughput sequencing of the 16 S rRNA gene (Fig. S3). From the shotgun data, both analyses showed that the taxonomic composition of the unassembled fraction was different from that of the assembled fraction. The use of phylogenetic marker genes highlighted differences in prokaryotic and viral compositions (Fig. 3). The analysis showed that the assembled fraction had one characteristic phylum, *Proteobacteria*. At the class level, *Rhizobiales* and *Betaproteobacteria* with *Burkholderiales* dominated this fraction. The unassembled community had a larger number of signature taxa, including *Verrucomicrobia*, *Actinobacteria*, *Bacteroidetes*, and *Thaumarchaeota*, within *Archaea*. Among this fraction *Proteobacteria*, *Gammaproteobacteria* dominated. Interestingly, this fraction was also characterized by viruses. Since, in this study, the microbial biomass was gathered on 0.2 µm pore-sized filters, viruses were possibly present as prophages or particles in the lytic phase. The ASVs from the amplicon sequencing were separated in two fractions based on an abundance threshold of 0.01% (Fig. S3). The abundant ASVs were dominated by the SAR11 clade whereas the rare ASVs were also more diverse as observed for unassembled metagenomic read fraction. In the rare ASV fraction, the *Gammaproteobacteria, Bacteroidetes Verrucomicrobia* and *Actinobacteria* were more common than in the abundant fraction. Finally, the two fractions based on the assembled/unassembled reads and the reference method for deciphering the rare biosphere based on a threshold (i.e. 0.01%) gave similar results (Fig. 3 and Fig. S3). We can hypothesize that the unassembled reads capture the majority of the rarest fraction of microorganisms.

### Identifying metabolic capabilities among the assembled and unassembled fraction

**Fig. 4:**
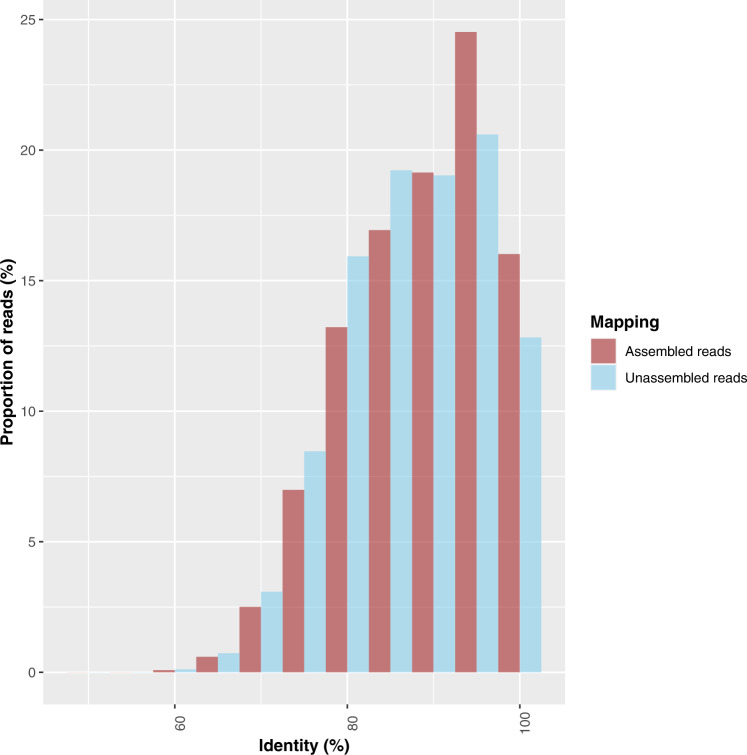
Distribution of the identities between assembled and unassembled reads against the UNIREF90 database. The assembled and unassembled reads were mapped against UNIREF90 database (results obtained from UNIREF100 were displayed in the supplementary materials Fig S5) by using DIAMOND [35] with the blastx mode.

The alignment data showed that for all sampling dates there was a higher proportion of reads that aligned to the UNIREF90 references in the assembled (44.1%) than unassembled fraction (38.5%) (Fig. S4). The overall percentage of aligned reads for both assembled and unassembled reads was low. In addition, a higher proportion of the assembled read alignments had high identity values than those of the unassembled reads (Fig. 4). When comparing both alignment scores and identities for UNIREF90 and UNIREF100, the differences between unassembled and assembled reads were highly significant (ANOVA two ways: assembled/unassembled × sampling dates; Fig. S5). The main factor explaining the variations in identity or scores was “mappability” against contigs and not sampling date.

**Fig. 5:**
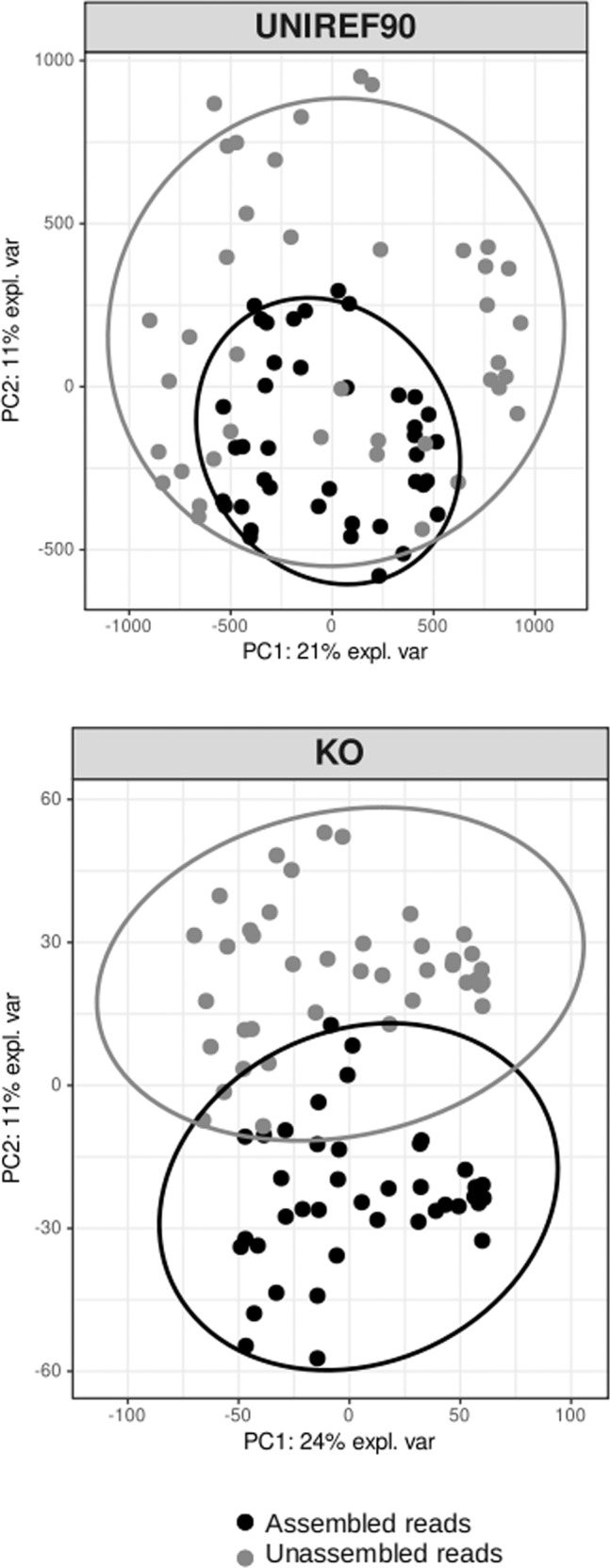
Sparse Principal Component Analysis conducted of the read composition annotated against the UNIREF90 (top) and the KO databases (bottom). The ANOSIM statistics based on the Bray-Curtis similarity were R = 0.63 (*P* < 0.01) for the UNIREF90 dataset and R = 0.90 (*P* < 0.01) for KO results.

The sparse principal component analysis (sPCA) based on UNIREF90 and KEGG annotated genes separated the assembled and unassembled fractions (Fig. 5). The multivariate analysis explained 31% (UNIREF90 clusters) and 36% (KEGG clusters) of the variance along axes 1 and 2. By comparing pathways (KO) present in the assembled *vs*. unassembled fractions, we identified two pathways involved in photosynthesis and flagellar assembly, which were enriched in the assembled communities (Fig. S6). The unassembled fraction was not significantly enriched in any of the pathways referenced in the KEGG database. This result is congruent with the previous statistical analysis showing few KOs enriched in this fraction (Table 1).

### Covarying gene groups of the assembled and unassembled communities

In total, 114 non-redundant CAGs were identified. The mean completeness was 53.19% (33.47–89.71) for the 56 uCAGs and 47.27% (30.25–80.07) for the 58 aCAGs. The mean contaminations were 4.44% and 4.06% for the uCAGs and aCAGs species, respectively. The uCAGs consisted of 65,787 genes and 59,470 genes for the aCAGs. The UNIREF proteins were linked to KEGG features to identify 3072 KOs in 78 CAGs. A total of 765 KOs specifically belonged to the uCAGs (37) and 2287 to the aCAGs (41).

Of the 125,257 genes (UNIRE90 + 16 S rRNA genes) found to be enriched in the unassembled fraction (Table 1), 16,878 were found in the uCAGs (13.4%). This proportion reached 14.7% for genes enriched in assembled fraction. Three CAGs contained 16 S rRNA genes that were found to be significantly enriched in the unassembled fraction (*Gammaproteobacteria*, *Flavobacteriia*, *and Betaproteobacteria*), and one CAG included a 16 S rRNA gene present exclusively in the unassembled reads during all sampling dates. This CAG belonged to *Alphaproteobacteria* (*Nisaea* genus).

### Key constituents in marine ecosystems deciphered by a network approach

**Fig. 6:**
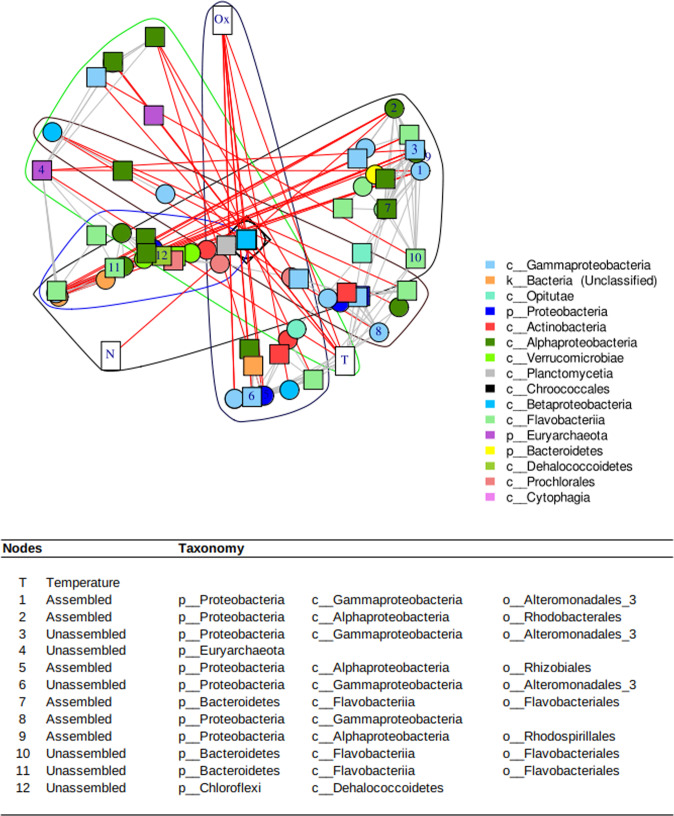
Network representation of the relationship between uCAG (square vertices), aCAG (circle) and physicochemical parameters (rectangle, T: temperature, Ox: oxygen and N: nitrite) and Louvain clusters. Red lines between nodes indicate negative Spearman correlations whereas gray edges correspond to positive correlations. The table below the graphics shows the best keystones in the network inferred from the « ExpectedInfluence » parameter (see Fig. S8). The numbers in the first column correspond to the numbering of the vertex in the network.

The network built with 49 uCAGs and 46 aCAGs was binned in 18 clusters (Louvain method), of which five had more than three vertices (CAGs or physico-chemical parameters). All of these large clusters included two kinds of CAGs and three were associated with physico-chemical parameters: temperature, oxygen, and nitrite concentration (Fig. 6 and Fig. S7). We identified the main metabolic pathways associated with each cluster by considering the pathways represented by at least 25% of the KEGG orthologs included in the pathway of interest. The major common pathways corresponded mainly to metabolisms involved in amino acid biosynthesis, but photosynthesis pathways also characterized one of these clusters (Fig. S7 - Cluster 17).

When analyzing the temporal dynamics of the CAGs, the spring and summer seasons determined their dynamics (Fig. S7). The network parameters allow us to decipher the main “influencers” or keystone species (Fig. 6), and temperature appears to be the main key parameter. Among the keystone species, uCAGs and aCAGs were present and mainly classified in the *Proteobacteria* phylum (*Alpha* and *Gammaproteobacteria*). Interestingly, *Archaea* classified as *Euryarchaeota* appeared in this top ranking.

## Discussion

In this paper we present an overview of the rare genomic content of marine microbial communities based on the reads “mappability” against contigs, and defined for the first time at the taxa or gene level. The congruence between the detection clade-specific marker genes in the assembled and unassembled reads (Fig. 3) and metabarcoding results (Fig. S3), separating abundant and rare microbes, indicates that the most part of the unassembled reads belonged to rare marine species. The unassembled reads could also have originated from strain heterogeneity manifested as single nucleotide variations and small insertions or deletions [4]. However, the assembler used in this paper takes into account the coverage ratios between adjacent edges in the assembly graph (*de Bruijn Graph*) to replace it with high-covered alternatives, and acts therefore as a consensus assembly reducing information about individual strains. As only the most abundant microbes are assembled by common bioinformatics tools [2, 3], and because the kind of assembler used performs poorly with strain heterogeneity, the unassembled reads that we focused on most certainly represent members of the rare biosphere.

### Community composition of the assembled and unassembled fractions

The comparison of the taxonomy inferred from metabarcoding in the abundant and rare fraction (<0.01%) with those deduced from phylogenetic markers included in assembled and unassembled reads, revealed similar patterns between the two approaches. The unassembled fraction, and the rare 16 S rRNA amplicons, were both characterized by a higher community diversity and by a higher abundance of *Gammaproteobacteria*, *Verrucomicrobia*, *Actinobacteria* and *Bacteroidetes*. The similarity between the two data sets is noteworthy since the approaches have different potential biases. Metabarcoding is hampered by well-known PCR bias and the cut-off definition of the rare biosphere is always arbitrary (0.01% here). To date, 16 S or 18 S rRNA based studies describing the rare biosphere have used a cut off, often ranging between <1% [49] and <0.01% [48], which originates from the rank-curve distribution of microbial communities that shows a long ‘tail’ of low abundance taxa [13]. In our metagenomic approach, the delineation between rare and abundant pool genes does not depend on an arbitrary cut off, but on sequencing depth and contig length. However, the delineation between rare and abundant may still depend on the sequencing effort. Our approach differs from an earlier metagenomics study that defined rare members as sequence assemblies being in the “tail” of the contig rank abundance curve, or ~0.005% in relative abundance [23]. The two methods that we used, metabarcoding and metagenomic based, allowed to detect the prokaryotes characterizing the abundant fraction, the *Alphaproteobacteria* phylum (SAR11 clade), which dominates marine bacteria [50]. Its ecological importance at our study site was underlined by the network analysis where it appeared among the main keystone taxa. Interestingly, the rare gene pool (unassembled data) was characterized by viruses. These viral genes detected mainly in the rare fraction corresponded likely to the replication of the DNA phage before the cell lysis. The rare community can therefore include some taxa under a strong selection pressure through viral lysis. Earlier experimental work suggested that some rare taxa may indeed have high susceptibility to viral attack [51]. This idea is, however, counter intuitive within the frame of the “kill the winner” hypothesis [52], which suggests that rare microorganisms, because they are not abundant, have a lower probability of encountering virus [53]. The link between predation and rare taxa is then rather seen as an evolutionary advantage for escaping top-down regulation [13]. Our data adds arguments for another hypothesis which suggests that lysis or predation are maintaining some particular taxa in a state of rarity.

### Seasonal dynamics and keystone species

Our study showed that the unassembled reads of metagenomes responded strongly to seasonal variations and corresponded certainly to an adaptation of the communities to specific environment conditions (light, temperature, nutrients etc…). This unassembled gene pool, which could correspond mostly to rare taxa as discussed above, displayed a reproducible pattern of temporal dynamics that was stronger than that of the assembled fraction, which in turn could represent the abundant microorganisms. The rare fraction thus showed a strong seasonal pattern for both similar and dissimilar communities (Fig. 2). Conversely, the rhythm of the abundant fraction (i.e. assembled reads) was noisier, with no patterns for communities sampled during opposite seasons. The abundant gene pool could thus correspond to core marine taxa with few temporal variations or to housekeeping genes. Thus, the overall seasonality of the microbial communities in response to the environment was mainly driven by the rare gene pool. A similar observation was made from coastal sands, where turnover in community composition was no longer observed when 50% of the rare species were removed from the dataset [54], and the Arctic Ocean where the rare biosphere was sensitive to environmental heterogeneity [55]. Rare communities can be classified according to different patterns of seasonal abundance and activity [17]. Within this classification, there is a group defined as rare taxa that never bloom but are active. It has been shown in bacteria, Archaea, and Eukaryotes [14, 17, 49]. These rare but active taxa also have a temporal pattern linked to biotic or abiotic parameters. Even though our metagenomics approach does not allow to infer activity, the reproducible seasonal dynamics of the continually rare community that we observed could suggest that they are active.

Overall, the binning step allowed the reconstruction of the main bacterial and archaeal phyla detected by the metaphlan pipeline (Fig. 3), with the exception of *Thaumarchaeota* (Table S2), and the network provided a good overview of the microbial interactions along the seasonal dynamics. Among the top “influencers” within this network were temperature, abundant microorganisms, and six rare taxa belonging to *Gammaproteobacteria*, *Flavobacteriia*, *Dehalocccoidetes*, and *Euryarchaeota*. The temperature had a significant influence on the microbial components of this network. Such result is not surprising, but it can be viewed as a validation of our approach. This influence is also noticeable at the read scale, since temporal variation was strongly associated with seasonality (Fig. 2). The link between heterotrophic bacterial metabolism and temperature is generally associated with nutrient availability, such as organic matter released from phytoplankton or grazing [56]. *Alphaproteobacteria* (*Rhodobacterales*) appeared twice in the top influencers, but were also challenged by other taxa, such as *Gammaproteobacteria* and *Bacteroidetes*. Arandia-Gorostidi et al. [57] showed that the growth of these taxa was strongly related to temperature changes, whereas *Alphaproteobacteria*, such as SAR11, showed the lowest temperature sensitivity [58]. The *Gammaproteobacteria* class, and more specifically the *Alteromonodales*, dominated the main influencers in this network. After *Alphaproteobacteria*, this class was the most abundant in ICoMM data [58] and *Alteromonodales*, such as *Oceanospirillales* or *Vibrionales*, contains mainly marine species. Therefore, *Alteromonas* could contribute significantly to the flux of dissolved organic carbon and nutrient mineralisation in the upper ocean [59, 60]. Furthermore, *Euryarchaeota* was also found to have a key role. The CAG built in this study does not allow for a precise taxonomy; however, a previous study on the same site highlighted the presence of the MGII clade [17, 61] now defined as an order lineage. The ecological success of the MGII group could be due to the presence of light-harvesting proteins (i.e. proteorhodopsin) [61–63]. Recently, the partially reconstructed MGIIa genome revealed the presence of glycoside hydrolases that are possibly involved in algal substrate breakdown [64, 65].

### Rare and abundant gene pools: many unknown functions

This study showed that there was significantly more unknown genes in the rare fraction than in the abundant fraction (Fig. 4 and Fig. S4). The microbial rare biosphere could thus be seen as a large pool of genes possessing known and unknown functions and considered a reservoir of “genetic novelty” [20, 66]. Since the rare gene pool showed strong temporal dynamics, it indicates that this reservoir of rare functions plays a role in ecosystem functioning. Some of the rare reads could nevertheless be mapped against database references (UNIREF or KEGG). They corresponded to known potential functions, but the identity of these rare genes was significantly lower than that of the abundant ones. This suggests that the rare gene pool harbors different variants of known genes found in abundant microbes. It should be noted that no metabolic pathways could be built from the identified rare KOs. The sequencing depth may have been too shallow to detect all the steps of the pathways present in the rare microbes, or some of the steps may be conducted by proteins coded by unknown genes.

For the abundant microorganisms, the fraction of the mapped reads against the UNIREF databases (90 or 100) always represented a low proportion of the total clean reads (<45%). This result at the short-read scale is in agreement with previous studies showing that 40%–60% of the coding genes cannot be assigned to a known function in the marine environment [67, 68]. Even in the human gut microbiome, which has been extensively studied, approximately 40% of the genes have unknown functions, although the “mappability” of the metagenomes against microbial genomes reaches ~80% [69]. The unmapped reads can correspond to new functions harbored by known lineages or the dark matter of unknown taxa [67]. Our results showed that little is known about the genes and their coded functions present in marine microbial communities. When analyzing known functions among abundant microbes, some metabolic pathways could be described, but they represented the most common metabolic pathways involved in primary metabolic processes, such as photosynthesis or flagellar assembly (Fig. S6).

## Conclusion

In this work, we show that the rare microbial gene pool of the marine environment is made of key species and represents a large number of potentially novel functions. In addition, based on the presence of viruses in the rare fraction, we hypothesized that the state of rarity could be maintained by viral lysis. However, the procedures used in this study were not dedicated to the detection of viruses and thus a large diversity may have escaped detection. A metagenomic based approach helps the challenging characterization of the members of the rare biosphere and promotes the discovery of new putative functions.

## Supplementary information

Supplemental material: legends

Supplemental material: Tables S1-S4

Supplemental material: Figures S1-S8

## Data Availability

Raw reads were archived in the ENA repository under accession number PRJEB26919.
