# Supplementary material for: Seasonal microbial dynamics in the ocean inferred from assembled and unassembled data: a view on the unknown biosphere
Source: ISME Commun. 2022 Sep 21;2:87. doi: 10.1038/s43705-022-00167-8 (PMC9723795; doi:10.1038/s43705-022-00167-8)
Supplement: Supplementary file 1 — Supplemental material: legends [file 43705_2022_167_MOESM1_ESM.pdf]

Supplementary materials

Fig S1. NMDS based on Bray Curtis dissimilarity computed from MetaFast separating assembled and unassembled microbial communities.

Fig S2. spca analysis of microbial communities based on 16S rRNA extracted from the assembled and unassembled fractions of the metagenomes.

Fig S3. Abundant (top) and rare (bottom) communities deciphered by metabarcoding.

Fig S4. Distribution of the assembled and unassembled reads aligned to the UNIREF90 (A) and UNIREF100 (B) databases.

Fig S5. Distribution of the scores and identities of the assembled and unassembled reads aligned to the UNIREF90 (A) and UNIREF100 (B) databases.

Fig S6. Photosynthesis and flagellar assembly pathways. Green rectangles corresponds to an enrichment in the assembled reads and red in the unassembled reads.

Fig S7 Temporal dynamics (z-scores) of the unassembled and assembled CAGs inside the main network clusters assessed by the Louvain methods. The clusters composed of less than 3 vertices are not represented. The grey rectangle represents spring and summer periods. The table displays the mains metabolic pathways in the clusters 16 and 17 (Any pathway with at least 25 % of the KOs were detected in the other clusters),

Fig S8. « ExpectedInfluence » parameter computed from the network with the package qgraph under R.

Table S1. Sampling date and environmental parameters

Table S2. Sequencing and main statistics.

Table S3. Effects of the cleaning procedures on the functional abundance tables

Table S4. Completeness, contamination and taxonomy of the CAGs built with the three methods described in the materials and methods (ass : assembled or aCAG - unass : unassembled or uCAG –

cano : Canopy method (Nielsen et al. 2014) - miner-msp : MSPminer method (Plaza Oñate et al. 2019) - mixo: new approach described in materials and methods section)
