# Supplementary material for: Seasonal microbial dynamics in the ocean inferred from assembled and unassembled data: a view on the unknown biosphere
Source: ISME Commun. 2022 Sep 21;2:87. doi: 10.1038/s43705-022-00167-8 (PMC9723795; doi:10.1038/s43705-022-00167-8)
Supplement: Supplementary file 2 — Supplemental material: Tables S1-S4 [file 43705_2022_167_MOESM2_ESM.pdf]

Table S1. Sampling date and environmental parameters

	Date	Temperature (°C)	Salinity (PSU)	Oxygen (mL/L)	pH	NH4	NO3 (µmol/L)	NO2 (µmol/L)	PO4 (µmol/L)	SIOH4 (µmol/L)	CHLA (µg/L)	Day Length (h)
Sample 1	03/01/2012	13.8	38.1	13.8	38.1	0.0	0.6	0.2	0.0	0.7	0.7	9.2
Sample 2	31/01/2012	11.8	38.0	11.8	38.0	0.0	1.6	0.4	0.0	0.3	0.3	10.0
Sample 3	21/02/2012	10.5	38.2	10.5	38.2	0.0	2.3	0.2	0.0	0.5	0.5	10.9
Sample 4	07/03/2012	10.9	38.2	10.9	38.2	0.1	1.1	0.1	0.1	0.8	0.8	11.6
Sample 5	13/03/2012	11.2	38.2	11.2	38.2	0.1	0.5	0.2	0.0	0.6	0.6	11.9
Sample 6	04/04/2012	13.8	37.9	13.8	37.9	0.0	0.4	0.1	0.0	0.3	0.3	12.9
Sample 7	23/04/2012	13.3	38.2	13.3	38.2	0.0	1.1	0.1	0.1	0.3	0.3	13.8
Sample 8	09/05/2012	15.5	36.6	15.5	36.6	0.0	0.9	0.1	0.1	1.8	1.8	14.4
Sample 9	07/06/2012	19.5	37.6	19.5	37.6	0.0	0.2	0.0	0.0	0.1	0.1	15.2
Sample 10	12/07/2012	20.1	37.9	20.1	37.9	0.0	0.0	0.0	0.0	0.1	0.1	15.0
Sample 11	06/08/2012	21.7	38.0	21.7	38.0	0.1	0.1	0.0	0.0	0.2	0.2	14.2
Sample 12	20/08/2012	22.9	38.2	22.9	38.2	0.1	0.1	0.0	0.0	0.2	0.2	13.7
Sample 13	22/10/2012	18.2	38.2	18.2	38.2	0.3	0.3	0.1	0.1	0.5	0.5	10.7
Sample 14	05/11/2012	16.6	37.9	16.6	37.9	0.2	0.6	0.1	0.1	0.6	0.6	10.1
Sample 15	19/11/2012	15.4	37.9	15.4	37.9	0.3	0.9	0.2	0.1	0.4	0.4	9.6
Sample 16	12/12/2012	13.1	38.0	13.1	38.0	0.0	1.4	0.2	0.0	0.5	0.5	9.1
Sample 17	15/01/2013	12.7	37.6	12.7	37.6	0.0	0.9	0.3	0.1	1.2	1.2	9.4
Sample 18	04/02/2013	11.1	38.1	11.1	38.1	0.0	1.8	0.3	0.1	1.2	1.2	10.1
Sample 19	11/03/2013	11.5	34.7	11.5	34.7	0.1	5.9	0.2	0.2	2.6	2.6	11.7
Sample 20	22/04/2013	13.1	37.1	13.1	37.1	0.2	1.8	0.2	0.0	0.5	0.5	13.7
Sample 21	06/05/2013	13.9	37.3	13.9	37.3	0.1	1.6	0.2	0.1	0.2	0.2	14.3
Sample 22	03/06/2013	15.0	37.8	15.0	37.8	0.0	0.2	0.0	0.1	1.4	1.4	15.1
Sample 23	01/07/2013	19.1	37.9	19.1	37.9	0.0	0.1	0.0	0.0	0.5	0.5	15.3
Sample 24	26/08/2013	22.6	37.9	22.6	37.9	0.1	0.2	0.0	0.0	0.1	0.1	13.4
Sample 25	28/10/2013	19.0	36.8	19.0	36.8	0.5	1.6	0.1	0.0	1.1	1.1	10.5
Sample 26	13/11/2013	16.5	37.9	16.5	37.9	0.1	0.2	0.0	0.0	1.0	1.0	9.9
Sample 27	12/12/2013	12.7	38.2	12.7	38.2	0.0	2.5	0.2	0.1	0.5	0.5	9.1
Sample 28	24/02/2014	12.7	38.0	5.7	8.1	0.1	1.7	0.3	0.1	2.5	0.7	11.0
Sample 29	07/04/2014	13.5	37.1	6.0	8.0	0.2	1.1	0.1	0.0	0.5	2.5	13.0
Sample 30	22/04/2014	15.2	37.4	5.5	8.2	0.0	0.1	0.1	0.0	0.8	1.2	13.7
Sample 31	10/06/2014	17.7	37.7	5.5	8.1	0.0	0.0	0.0	0.0	0.8	0.3	15.2
Sample 32	21/07/2014	20.5	37.9	5.0	8.1	0.0	0.2	0.0	0.0	2.8	0.3	14.8
Sample 33	04/08/2014	22.0	37.8	4.2	8.1	0.0	0.2	0.0	0.0	0.7	0.3	14.4
Sample 34	01/09/2014	21.7	37.9	5.1	8.6	0.0	0.0	0.1	0.0	0.5	0.2	13.2
Sample 35	12/11/2014	18.2	38.1	5.0	8.1	0.1	0.3	0.1	0.0	1.0	0.3	9.9
Sample 36	24/11/2014	17.3	37.7	5.3	8.1	0.4	0.7	0.1	0.2	1.2	0.6	9.5
Sample 37	08/12/2014	16.2	37.5	5.5	8.1	0.2	1.4	0.3	0.0	3.6	0.3	9.2
Sample 38	08/01/2015	13.3	37.8	5.8	8.1	0.1	0.5	0.3	0.0	1.8	0.7	9.3
Sample 39	22/01/2015	12.7	37.8	5.9	8.1	0.1	0.3	0.2	0.0	1.2	1.1	9.6
Sample 40	02/02/2015	12.6	38.1	5.8	8.2	0.1	1.4	0.2	0.0	3.8	0.6	10.0

Table S2. Sequencing and main statistics.

							UNIREF 90		UNIREF100		KO		SILVA	
**Sample**	**Raw reads**	**HQ reads**	**Contigs ≥ 1kb**	**Total contig length (nt)**	**unmapped reads**	**mapped reads**	**unmapped reads**	**mapped reads**	**unmapped reads**	**mapped reads**	**unmapped reads**	**mapped reads**	**unmapped reads**	**mapped reads**
Sample 1	59273132	57958251	41244	98262145	44593508	13364743	17744984	6314543	14117390	5010681	6766110	2603287	27983	4990
Sample 2	80826740	79163692	48367	123062579	62841116	16322576	25548060	7813758	21145099	6415748	10024110	3513576	31369	4955
Sample 3	71312636	69857457	41513	111756344	54261976	15595481	22433056	7568176	18701570	6361829	9401751	3708808	29211	4519
Sample 4	80636430	79362166	39939	109512619	63381770	15980396	23922649	6976595	20328038	5995649	11540450	3655534	31165	3547
Sample 5	95291086	93131454	56595	169734749	70714308	22417146	27075890	9352797	22942805	7941408	12795658	4812572	26767	4591
Sample 6	52835444	51942847	35548	98399996	40060850	11881997	14437818	4926161	11961467	4159561	6874015	2527812	20607	3128
Sample 7	51079736	49380567	40508	102758931	38149046	11231521	15608984	5234680	12979380	4352408	6588399	2374903	26193	4676
Sample 8	73445534	71280727	49778	126501323	53386528	17894199	16974682	5948624	13877432	5018846	7253899	2796688	19265	3707
Sample 9	90753470	87683664	48018	126073118	66472599	21211065	23456209	7412645	19771960	6479531	10146005	3388588	27009	7245
Sample 10	104860772	102293843	57819	179149121	77392016	24901827	27983866	9789154	23196981	8293394	12343189	4593072	18863	4016
Sample 11	74392204	71640388	52397	156895851	55492119	16148269	22447996	7455555	18604685	6349977	10385948	3648889	31133	6080
Sample 12	34871574	33772351	31070	76907204	26176962	7595389	10468840	3530427	8736353	3035491	4846031	1726263	14469	2821
Sample 13	72532844	70335589	54369	140748920	54403140	15932449	20873638	7298011	16941868	5980003	8151361	2964819	25543	4931
Sample 14	74872672	72846496	61964	149954009	55958010	16888486	20720685	7510087	16743569	6055242	8069160	3116919	25498	4894
Sample 15	64281530	63027247	53199	120289337	47399605	15627642	18932849	7417287	15201591	5968001	7377584	3093858	23716	4616
Sample 16	106010408	102302513	61926	145187693	78377597	23924916	27240211	10227759	21443828	8207855	10600300	4477385	34273	6052
Sample 17	100810750	98495009	49889	116274315	76504402	21990607	27366704	9556273	22243394	7808796	11608378	4477537	32466	5889
Sample 18	48838402	47515963	37698	89710208	36673413	10842550	16314197	5474541	13579889	4571057	6560683	2516221	21729	3732
Sample 19	46597854	44964848	23994	70893350	35466177	9498671	11144115	3249687	9957275	2894784	6093029	1738209	17482	4302
Sample 20	73729860	71469602	52630	148504862	55238042	16231560	22624288	7468118	18546850	6220009	10637098	3801247	29936	5649
Sample 21	65923978	63996584	44790	145087132	49337751	14658833	19797958	6278121	16556082	5218864	8858459	2838609	26232	7022
Sample 22	58442788	56487206	44179	112636265	44041597	12445609	19554352	5699133	16193963	4777311	8311466	2566026	23809	4256
Sample 23	98347746	95156563	41390	122193270	75858100	19298463	26378686	7150324	21900089	6173804	11792198	3420789	29423	8161
Sample 24	84345622	81841740	50854	161740082	62989261	18852479	23712351	7887602	19228848	6547768	10639223	3693963	30358	7162
Sample 25	60805948	59212336	52256	148645942	44086255	15126081	17889251	6636137	14403477	5384308	7311577	2801849	20559	5512
Sample 26	64913662	63090391	58835	140734121	47145774	15944617	19257860	7558899	15567740	6110545	7886946	3220050	25614	5743
Sample 27	68579126	66588511	44217	105396708	50939677	15648834	19738834	7581930	15372615	6041119	7432843	3202869	25946	5904
Sample 28	79561348	77673692	58673	142607160	59188672	18485020	23569642	9061095	19009395	7386984	9301854	4013124	29089	6043
Sample 29	71354090	69881637	61803	169475995	52749658	17131979	21761777	7349253	18072931	6109384	9626090	3291964	25882	4104
Sample 30	113230060	110798145	51209	156651985	88814754	21983391	28957198	8097672	23718462	6667758	13703444	4004660	33717	6290
Sample 31	114367198	111753381	68044	186932189	86065519	25687862	33574233	11208336	27891691	9520448	15782159	5630437	31003	4906
Sample 32	54582720	52700198	44532	110010378	40640046	12060152	14851597	5027511	12009786	4189769	6521950	2364754	17793	3138
Sample 33	68653980	66202174	57680	155231185	49950779	16251395	21268390	7754553	17368462	6598308	9830173	3873255	30580	4946
Sample 34	70368772	68587662	58720	166717960	50882440	17705222	21513514	8236950	17481664	6889401	9275249	3706257	26127	5610
Sample 35	74795486	72529047	67151	143637806	54130612	18398435	20988244	8631233	16646564	6876798	8052044	3460407	28350	5386
Sample 36	107935088	105270469	88088	220361755	76296829	28973640	27344301	12150638	22016656	9790225	10592706	4766742	34635	8128
Sample 37	74502570	72806977	60035	142623448	54108954	18698023	19647035	8813076	15561830	6547609	7226123	3247426	24875	6020
Sample 38	76479148	74514541	57514	134746061	57119117	17395424	20706268	7716631	16547788	6095887	7811834	3129854	28238	5325
Sample 39	69334770	68072943	49362	130291696	52196745	15876198	21793932	7646863	17591673	6120515	8854864	3325714	27517	4641
Sample 40	50666858	48966052	33115	75897135	37767712	11198340	14552988	5307755	11578548	4217459	5623197	2297260	19865	3619

Table S3. Effects of the cleaning procedures on the functional abundance tables

	Before cleaning		After cleaning	
Databases	Features	Reads	Features	Reads
**SILVA**	6959	1260545	846	1191644
**KEGG**	9826	4.97E+08	6984	4.97E+08
**uniref90**	7725889	1.15E+09	1210645	1.09E+09
**Uniref100**	8471020	9.40E+08	NA	NA

Table S4. Completeness, contamination and taxonomy of the CAGs built with the three methods described in the materials and methods.
(ass : assembled or aCAG - unass : unassembled or uCAG – cano : Canopy method (Nielsen et al. 2014) - miner-msp : MSPminer method (Plaza Oñate et al. 2019) – mixo: new approach described in materials and methods section)

CAGs	Completeness	Contamination	Kingdom	Phylum	Class	Order	Family	Genus	Species
ass-cano-CAG0026	61.2	4.13	k__Bacteria	p__Proteobacteria	c__Alphaproteobacteria	o__Rhodospirillales	f__Rhodospirillaceae	g__Nisaea	
ass-cano-CAG0035	46.22	0.84	k__Bacteria	p__Proteobacteria	c__Alphaproteobacteria	o__Rhodobacterales	f__Rhodobacteraceae		
ass-cano-CAG0040	40.47	5.17	k__Bacteria	p__Proteobacteria	c__Gammaproteobacteria				
ass-cano-CAG0045	49.54	1.01	k__Bacteria	p__Proteobacteria	c__Alphaproteobacteria	o__Rhodospirillales	f__Rhodospirillaceae		
ass-cano-CAG0048	35.66	0	k__Bacteria	p__Proteobacteria	c__Gammaproteobacteria				
ass-cano-CAG0049	49.34	9.44	k__Bacteria	p__Proteobacteria	c__Gammaproteobacteria	o__Alteromonadales_3	f__Alteromonadaceae		
ass-cano-CAG0050	34.85	1.72	k__Bacteria	p__Proteobacteria	c__Gammaproteobacteria	o__Alteromonadales_3	f__Alteromonadaceae		
ass-cano-CAG0051	52.59	0.92	k__Bacteria	p__Proteobacteria	c__Alphaproteobacteria	o__Rhodobacterales	f__Rhodobacteraceae		
ass-cano-CAG0056	35.75	5.17	k__Bacteria	p__Verrucomicrobia	c__Verrucomicrobiae	o__Verrucomicrobiales	f__Verrucomicrobiaceae		
ass-cano-CAG0059	51.77	7.78	k__Bacteria	p__Bacteroidetes	c__Flavobacteriia	o__Flavobacteriales	f__Flavobacteriaceae		
ass-cano-CAG0060	53.72	5.78	k__Bacteria	p__Actinobacteria	c__Actinobacteria				
ass-cano-CAG0061	36.26	5.13	k__Bacteria	p__Proteobacteria	c__Alphaproteobacteria	o__Sphingomonadales	f__Sphingomonadaceae_3	g__Sphingobium	
ass-cano-CAG0064	67.45	9.1	k__Bacteria	p__Proteobacteria	c__Gammaproteobacteria				
ass-cano-CAG0066	52.26	1.63	k__Bacteria	p__Cyanobacteria	c__Chroococcales	o__Chroococcales	f__Cyanobium		
ass-cano-CAG0068	47.53	1.83	k__Bacteria	p__Verrucomicrobia	c__Opitutae	o__Opitutales			
ass-cano-CAG0069	43.1	0	k__Bacteria	p__Proteobacteria	c__Gammaproteobacteria	o__Alteromonadales_3	f__Alteromonadaceae		
ass-cano-CAG0070	70.2	1.92	k__Bacteria	p__Cyanobacteria	c__Prochlorales	o__Prochlorales	f__Prochlorococcaceae	g__Prochlorococcus	s__Prochlorococcus_marinus
ass-cano-CAG0072	44.45	9.47	k__Bacteria	p__Proteobacteria	c__Alphaproteobacteria	o__Rhodospirillales	f__Rhodospirillaceae		
ass-cano-CAG0073	49.49	4.01	k__Bacteria	p__Proteobacteria	c__Alphaproteobacteria	o__Rhodobacterales			
ass-cano-CAG0075	40.11	3.51	k__Bacteria	p__Bacteroidetes	c__Flavobacteriia	o__Flavobacteriales	f__Flavobacteriaceae		
ass-cano-CAG0077	58.97	0	k__Bacteria	p__Bacteroidetes	c__Flavobacteriia	o__Flavobacteriales			
ass-cano-CAG0078	61.33	7.91	k__Bacteria	p__Proteobacteria	c__Gammaproteobacteria				
ass-cano-CAG0081	39.87	3.79	k__Bacteria	p__Cyanobacteria	c__Prochlorales	o__Prochlorales	f__Prochlorococcaceae	g__Prochlorococcus	s__Prochlorococcus_marinus
ass-cano-CAG0088	37.23	1.72	k__Bacteria	p__Bacteroidetes	c__Flavobacteriia	o__Flavobacteriales	f__Flavobacteriaceae		
ass-cano-CAG0089	47.85	9.63	k__Bacteria	p__Proteobacteria	c__Betaproteobacteria	o__Methylophilales			
ass-cano-CAG0091	31.27	0	k__Bacteria	p__Actinobacteria	c__Actinobacteria				
ass-cano-CAG0093	39.47	0	k__Bacteria	p__Proteobacteria					
ass-cano-CAG0104	43.36	5.18	k__Bacteria	p__Proteobacteria	c__Alphaproteobacteria				
ass-cano-CAG0108	31.99	0.54	k__Bacteria	p__Bacteroidetes	c__Flavobacteriia	o__Flavobacteriales			
ass-cano-CAG0109	46.33	0.66	k__Bacteria	p__Proteobacteria	c__Betaproteobacteria	o__Methylophilales			
ass-cano-CAG0114	35.8	0.74	k__Bacteria	p__Bacteroidetes	c__Flavobacteriia	o__Flavobacteriales			
ass-miner-msp_023	58.08	3.86	k__Bacteria	p__Proteobacteria	c__Alphaproteobacteria	o__Rhodobacterales	f__Rhodobacteraceae		
ass-miner-msp_025	80.07	4.92	k__Bacteria	p__Proteobacteria	c__Alphaproteobacteria	o__Rhodobacterales	f__Rhodobacteraceae		
ass-miner-msp_036	50.17	6.31	k__Bacteria	p__Verrucomicrobia	c__Verrucomicrobiae	o__Verrucomicrobiales	f__Verrucomicrobiaceae		
ass-miner-msp_037	47.87	6.49	k__Bacteria	p__Verrucomicrobia	c__Verrucomicrobiae	o__Verrucomicrobiales	f__Verrucomicrobiaceae		
ass-miner-msp_042	39.09	6.53	k__Bacteria	p__Proteobacteria	c__Gammaproteobacteria	o__Alteromonadales_3	f__Alteromonadaceae		
ass-miner-msp_049	41.18	1.72	k__Bacteria	p__Proteobacteria	c__Gammaproteobacteria				
ass-miner-msp_054	32.47	0	k__Bacteria	p__Proteobacteria	c__Alphaproteobacteria	o__Rhodospirillales	f__Rhodospirillaceae		
ass-miner-msp_056	55.25	2.3	k__Bacteria	p__Planctomycetes	c__Planctomycetia	o__Planctomycetales	f__Planctomycetaceae		
ass-miner-msp_058	49.48	1.72	k__Bacteria	p__Proteobacteria	c__Alphaproteobacteria	o__Rhodobacterales	f__Rhodobacteraceae		
ass-miner-msp_059	58.37	3.53	k__Bacteria	p__Cyanobacteria	c__Chroococcales	o__Chroococcales	f__Cyanobium		
ass-miner-msp_060	30.83	0	k__Bacteria	p__Proteobacteria	c__Gammaproteobacteria				
ass-miner-msp_064	77.2	6.81	k__Bacteria	p__Actinobacteria	c__Actinobacteria	o__Actinomycetales	f__Microbacteriaceae		
ass-miner-msp_068	55.68	9.55	k__Bacteria	p__Proteobacteria	c__Betaproteobacteria	o__Burkholderiales			
ass-miner-msp_073	68.23	1.97	k__Bacteria	p__Bacteroidetes	c__Flavobacteriia	o__Flavobacteriales			
ass-miner-msp_074	67.28	3.89	k__Bacteria	p__Cyanobacteria	c__Prochlorales	o__Prochlorales	f__Prochlorococcaceae	g__Prochlorococcus	s__Prochlorococcus_marinus
ass-miner-msp_079	36.04	1.73	k__Bacteria	p__Proteobacteria					
ass-miner-msp_082	54.77	2	k__Bacteria	p__Proteobacteria					
ass-miner-msp_083	38.01	0.78	k__Bacteria	p__Proteobacteria	c__Gammaproteobacteria				
ass-miner-msp_088	36.36	0	k__Bacteria	p__Proteobacteria	c__Gammaproteobacteria	o__Alteromonadales_3	f__Alteromonadaceae		
ass-miner-msp_089	32.4	0.93	k__Archaea	p__Euryarchaeota					
ass-miner-msp_090	50.11	0.54	k__Bacteria	p__Bacteroidetes	c__Flavobacteriia	o__Flavobacteriales	f__Cryomorphaceae		
ass-miner-msp_092	36.21	3.45	k__Bacteria	p__Actinobacteria	c__Actinobacteria				
ass-miner-msp_094	35.42	1.72	k__Bacteria	p__Bacteroidetes	c__Flavobacteriia	o__Flavobacteriales	f__Flavobacteriaceae		
ass-miner-msp_096	40.61	5.31	k__Bacteria	p__Proteobacteria	c__Alphaproteobacteria	o__Rhodospirillales	f__Rhodospirillaceae		

ass-miner-msp_099	55.8	3.81	k__Bacteria	p__Bacteroidetes				
ass-miner-msp_100	52.77	0.54	k__Bacteria	p__Bacteroidetes	c__Flavobacteriia	o__Flavobacteriales		
ass-miner-msp_103	30.25	0	k__Bacteria	p__Bacteroidetes	c__Flavobacteriia	o__Flavobacteriales		
ass-miner-msp_106	33.56	1.58	k__Bacteria	p__Actinobacteria	c__Actinobacteria	o__Actinomycetales		
ass-miner-msp_111	35.93	1.16	k__Bacteria	p__Bacteroidetes	c__Flavobacteriia	o__Flavobacteriales	f__Flavobacteriaceae	
ass-miner-msp_112	48.02	2.94	k__Bacteria	p__Bacteroidetes	c__Flavobacteriia	o__Flavobacteriales		
ass-miner-msp_113	38.09	4.39	k__Bacteria	p__Proteobacteria	c__Alphaproteobacteria	o__Rhizobiales		
ass-miner-msp_117	48.4	8.97	k__Bacteria	p__Proteobacteria	c__Betaproteobacteria	o__Methylophilales		
ass-miner-msp_118	47.1	3.23	k__Bacteria	p__Bacteroidetes	c__Flavobacteriia	o__Flavobacteriales		
ass-miner-msp_119	35.44	4.8	k__Bacteria					
ass-miner-msp_123	54.44	1.17	k__Bacteria	p__Proteobacteria	c__Betaproteobacteria	o__Methylophilales		
ass-miner-msp_129	31.5	0	k__Bacteria	p__Proteobacteria	c__Alphaproteobacteria	o__Rhodospirillales	f__Rhodospirillaceae	
ass-miner-msp_134	46.09	5.05	k__Bacteria	p__Bacteroidetes	c__Flavobacteriia	o__Flavobacteriales		
ass-miner-msp_139	32.26	1.53	k__Bacteria	p__Proteobacteria	c__Gammaproteobacteria	o__Alteromonadales_3	f__Alteromonadaceae	
ass-miner-msp_140	41.45	0.68	k__Bacteria	p__Verrucomicrobia	c__Opitutae	o__Opitutales		
ass-mixo-msp_236	39.77	9.32	k__Bacteria	p__Proteobacteria	c__Gammaproteobacteria	o__Pseudomonadales	f__Moraxellaceae	
ass-mixo-msp_318	51.63	4.81	k__Bacteria	p__Planctomycetes	c__Planctomycetia	o__Planctomycetales	f__Planctomycetaceae	
ass-mixo-msp_34	45.3	9.79	k__Bacteria					
ass-mixo-msp_349	39.55	8.82	k__Bacteria	p__Verrucomicrobia	c__Opitutae	o__Opitutales		
ass-mixo-msp_352	48.95	7.73	k__Bacteria	p__Proteobacteria	c__Alphaproteobacteria	o__Rhodospirillales	f__Rhodospirillaceae	
ass-mixo-msp_444	34.5	3.66	k__Bacteria	p__Proteobacteria				
ass-mixo-msp_502	50.91	4.52	k__Bacteria	p__Proteobacteria				
ass-mixo-msp_83	42.16	1.62	k__Bacteria	p__Actinobacteria	c__Actinobacteria	o__Actinomycetales		
unass-cano-CAG0027	82.18	5.73	k__Bacteria	p__Proteobacteria	c__Alphaproteobacteria	o__Rhodobacterales	f__Rhodobacteraceae	
unass-cano-CAG0032	84.12	6.73	k__Bacteria	p__Proteobacteria	c__Gammaproteobacteria			
unass-cano-CAG0048	77.79	6.87	k__Bacteria	p__Proteobacteria	c__Gammaproteobacteria			
unass-cano-CAG0050	82.27	4.93	k__Bacteria	p__Proteobacteria	c__Alphaproteobacteria	o__Rhodobacterales	f__Rhodobacteraceae	
unass-cano-CAG0052	67.08	2.72	k__Bacteria	p__Verrucomicrobia	c__Verrucomicrobiae	o__Verrucomicrobiales	f__Verrucomicrobiaceae	
unass-cano-CAG0056	79.6	6.83	k__Bacteria	p__Proteobacteria	c__Alphaproteobacteria	o__Rhodobacterales	f__Rhodobacteraceae	
unass-cano-CAG0062	58.16	7.67	k__Bacteria	p__Proteobacteria	c__Gammaproteobacteria	o__Alteromonadales_3	f__Alteromonadaceae	
unass-cano-CAG0066	73.66	7.03	k__Bacteria	p__Bacteroidetes	c__Flavobacteriia	o__Flavobacteriales	f__Cryomorphaceae	
unass-cano-CAG0073	50.36	1.94	k__Bacteria	p__Proteobacteria	c__Alphaproteobacteria	o__Rhodobacterales	f__Rhodobacteraceae	
unass-cano-CAG0074	72.06	2.64	k__Bacteria	p__Bacteroidetes	c__Flavobacteriia	o__Flavobacteriales	f__Flavobacteriaceae	
unass-cano-CAG0077	73.92	2.81	k__Bacteria	p__Bacteroidetes	c__Flavobacteriia	o__Flavobacteriales	f__Flavobacteriaceae	
unass-cano-CAG0080	61.47	7.28	k__Archaea	p__Euryarchaeota				
unass-cano-CAG0082	39.14	6.96	k__Bacteria	p__Proteobacteria	c__Gammaproteobacteria	o__Alteromonadales_3	f__Alteromonadaceae	
unass-cano-CAG0085	50.57	6.29	k__Bacteria	p__Bacteroidetes	c__Flavobacteriia	o__Flavobacteriales		
unass-cano-CAG0088	52.33	5.39	k__Bacteria	p__Proteobacteria	c__Gammaproteobacteria			
unass-cano-CAG0089	74.99	4.04	k__Bacteria	p__Bacteroidetes	c__Flavobacteriia	o__Flavobacteriales		
unass-cano-CAG0090	52.06	8.09	k__Bacteria	p__Bacteroidetes	c__Flavobacteriia	o__Flavobacteriales		
unass-cano-CAG0091	57.75	1.74	k__Bacteria	p__Bacteroidetes	c__Flavobacteriia	o__Flavobacteriales		
unass-cano-CAG0095	50.34	0	k__Bacteria	p__Proteobacteria	c__Alphaproteobacteria	o__Rhodospirillales	f__Rhodospirillaceae	
unass-cano-CAG0098	78.78	1.37	k__Bacteria	p__Proteobacteria	c__Betaproteobacteria	o__Methylophilales		
unass-cano-CAG0102	41.88	2.15	k__Bacteria	p__Bacteroidetes	c__Flavobacteriia	o__Flavobacteriales		
unass-cano-CAG0106	40.24	3.74	k__Archaea	p__Euryarchaeota				
unass-cano-CAG0108	53.06	1.35	k__Bacteria	p__Verrucomicrobia	c__Opitutae	o__Opitutales		
unass-cano-CAG0109	77.05	1.35	k__Bacteria	p__Verrucomicrobia	c__Opitutae	o__Opitutales		
unass-cano-CAG0113	34.5	0.18	k__Bacteria	p__Bacteroidetes	c__Cytophagia	o__Cytophagales	f__Cytophagaceae_2	
unass-cano-CAG0114	43.47	5.17	k__Bacteria	p__Proteobacteria	c__Gammaproteobacteria			
unass-cano-CAG0115	37.48	1.61	k__Bacteria	p__Bacteroidetes	c__Flavobacteriia	o__Flavobacteriales		
unass-cano-CAG0116	38.97	0.27	k__Bacteria	p__Actinobacteria	c__Actinobacteria	o__Actinomycetales		
unass-cano-CAG0117	46.77	0	k__Bacteria	p__Bacteroidetes	c__Flavobacteriia	o__Flavobacteriales		
unass-cano-CAG0124	36.64	4.76	k__Bacteria	p__Proteobacteria	c__Alphaproteobacteria	o__Rhodobacterales	f__Hyphomonadaceae	g__Oceanicaulis
unass-cano-CAG0133	42.31	0.91	k__Bacteria	p__Proteobacteria	c__Gammaproteobacteria	o__Thiotrichales		
unass-cano-CAG0136	37.2	3.45	k__Bacteria	p__Proteobacteria	c__Gammaproteobacteria	o__Legionellales		
unass-cano-CAG0145	36.13	3.45	k__Bacteria	p__Proteobacteria	c__Alphaproteobacteria	o__Rhodospirillales	f__Rhodospirillaceae	
unass-miner-msp_030	85.08	6.95	k__Bacteria	p__Proteobacteria	c__Betaproteobacteria	o__Burkholderiales		
unass-miner-msp_033	81.93	8.22	k__Bacteria	p__Proteobacteria	c__Gammaproteobacteria			
unass-miner-msp_045	76.68	5.44	k__Bacteria	p__Verrucomicrobia	c__Verrucomicrobiae	o__Verrucomicrobiales	f__Verrucomicrobiaceae	
unass-miner-msp_060	38.56	1.72	k__Bacteria	p__Proteobacteria	c__Gammaproteobacteria	o__Alteromonadales_3	f__Alteromonadaceae	

unass-miner-msp_063	35.75	2.6	k__Bacteria	p__Planctomycetes	c__Planctomycetia	o__Planctomycetales	f__Planctomycetaceae		
unass-miner-msp_082	81.53	9.22	k__Bacteria	p__Bacteroidetes	c__Flavobacteriia	o__Flavobacteriales			
unass-miner-msp_083	56.05	1.96	k__Bacteria	p__Proteobacteria	c__Alphaproteobacteria	o__Rhodospirillales	f__Rhodospirillaceae		
unass-miner-msp_086	38.71	5.17	k__Bacteria	p__Proteobacteria	c__Alphaproteobacteria	o__Rhodobacterales			
unass-miner-msp_087	79.74	8.77	k__Bacteria	p__Actinobacteria	c__Actinobacteria	o__Actinomycetales	f__Microbacteriaceae		
unass-miner-msp_088	86.88	3.32	k__Bacteria	p__Proteobacteria	c__Betaproteobacteria	o__Methylophilales			
unass-miner-msp_093	38.9	1.72	k__Bacteria	p__Proteobacteria					
unass-miner-msp_096	43.6	1.08	k__Bacteria	p__Bacteroidetes	c__Flavobacteriia	o__Flavobacteriales			
unass-miner-msp_099	52.27	2.56	k__Bacteria	p__Proteobacteria	c__Alphaproteobacteria	o__Rhodobacterales_2	f__Hyphomonadaceae		
unass-miner-msp_101	47.73	0.81	k__Bacteria	p__Actinobacteria	c__Actinobacteria	o__Actinomycetales			
unass-miner-msp_102	58.34	9.22	k__Bacteria	p__Proteobacteria	c__Gammaproteobacteria				
unass-miner-msp_105	79.63	5.77	k__Bacteria	p__Verrucomicrobia	c__Opitutae	o__Opitutales			
unass-miner-msp_107	50.87	4.5	k__Bacteria	p__Proteobacteria	c__Alphaproteobacteria	o__Rhodobacterales	f__Hyphomonadaceae	g__Oceanicaulis	
unass-miner-msp_109	58.94	0.75	k__Bacteria	p__Bacteroidetes	c__Flavobacteriia	o__Flavobacteriales			
unass-miner-msp_110	52.59	3.75	k__Bacteria	p__Bacteroidetes	c__Flavobacteriia	o__Flavobacteriales			
unass-miner-msp_111	40.83	5.67	k__Bacteria	p__Cyanobacteria	c__Prochlorales	o__Prochlorales	f__Prochlorococcaceae	g__Prochlorococcus	s__Prochlorococcus_marinus
unass-miner-msp_112	33.65	0	k__Bacteria	p__Bacteroidetes	c__Cytophagia	o__Cytophagales	f__Cytophagaceae_2		
unass-miner-msp_119	48.64	0	k__Bacteria	p__Bacteroidetes	c__Flavobacteriia	o__Flavobacteriales			
unass-miner-msp_130	34.94	4.79	k__Bacteria						
unass-miner-msp_132	41.98	8.2	k__Bacteria	p__Proteobacteria					
unass-miner-msp_146	34.83	3.2	k__Bacteria	p__Proteobacteria	c__Gammaproteobacteria	o__Legionellales			
unass-miner-msp_155	33.65	1.75	k__Bacteria						
unass-mixo-msp_122	40.22	4.86	k__Bacteria	p__Bacteroidetes	c__Flavobacteriia	o__Flavobacteriales	f__Cryomorphaceae		
unass-mixo-msp_144	45.54	5.62	k__Bacteria	p__Bacteroidetes	c__Flavobacteriia	o__Flavobacteriales	f__Cryomorphaceae		
unass-mixo-msp_309	40.04	9.44	k__Bacteria	p__Chloroflexi	c__Dehalococcoidetes				
unass-mixo-msp_355	33.47	0.72	k__Bacteria	p__Proteobacteria	c__Alphaproteobacteria				
unass-mixo-msp_405	34.04	3.57	k__Bacteria	p__Proteobacteria	c__Alphaproteobacteria	o__Rhodospirillales	f__Rhodospirillaceae	g__Nisaea	
unass-mixo-msp_448	36.85	1.72	k__Bacteria	p__Proteobacteria	c__Alphaproteobacteria	o__Rhodospirillales	f__Rhodospirillaceae		
unass-mixo-msp_516	89.71	6.67	k__Bacteria	p__Proteobacteria	c__Betaproteobacteria	o__Methylophilales			
unass-mixo-msp_610	43.94	5.54	k__Bacteria	p__Bacteroidetes	c__Flavobacteriia	o__Flavobacteriales			
unass-mixo-msp_654	45.7	6.91	k__Bacteria	p__Proteobacteria	c__Gammaproteobacteria	o__Alteromonadales_3	f__Alteromonadaceae		
unass-mixo-msp_677	42.22	7.25	k__Bacteria	p__Proteobacteria	c__Gammaproteobacteria				
unass-mixo-msp_679	37.74	5.62	k__Bacteria	p__Proteobacteria	c__Gammaproteobacteria				
unass-mixo-msp_71	36.32	5.13	k__Bacteria	p__Actinobacteria	c__Actinobacteria				
