# Supplementary material for: Seasonal microbial dynamics in the ocean inferred from assembled and unassembled data: a view on the unknown biosphere
Source: ISME Commun. 2022 Sep 21;2:87. doi: 10.1038/s43705-022-00167-8 (PMC9723795; doi:10.1038/s43705-022-00167-8)
Supplement: Supplementary file 3 — Supplemental material: Figures S1-S8 [file 43705_2022_167_MOESM3_ESM.pdf]

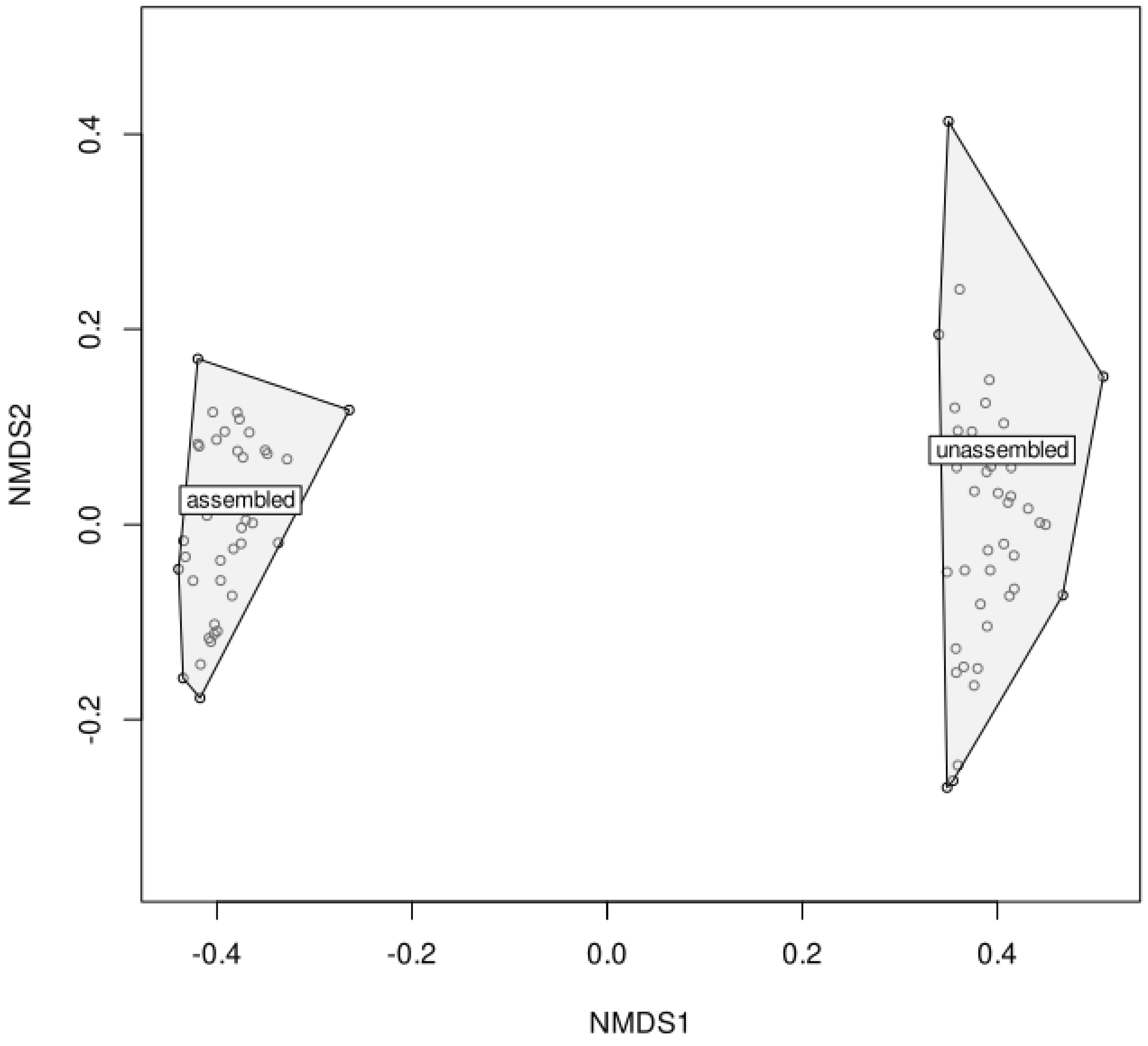

Fig S1. NMDS based on Bray Curtis dissimilarity computed from MetaFast separating assembled and unassembled microbial communities.

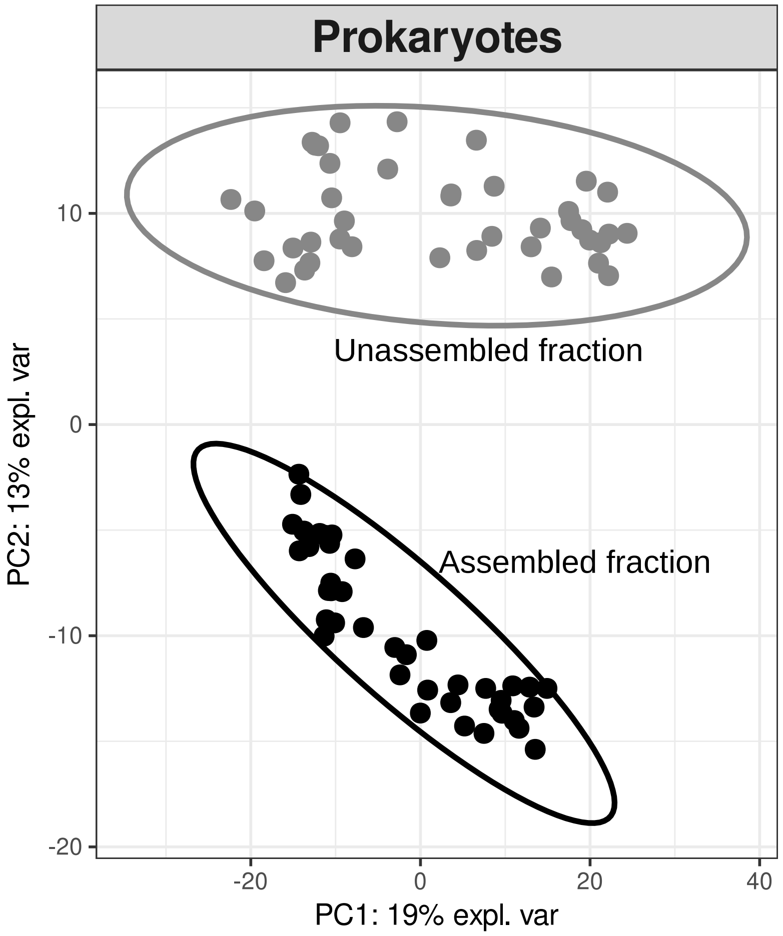

Fig S2. spca analysis of microbial communities based on 16S rRNA extracted from the assembled and unassembled fractions of the metagenomes.

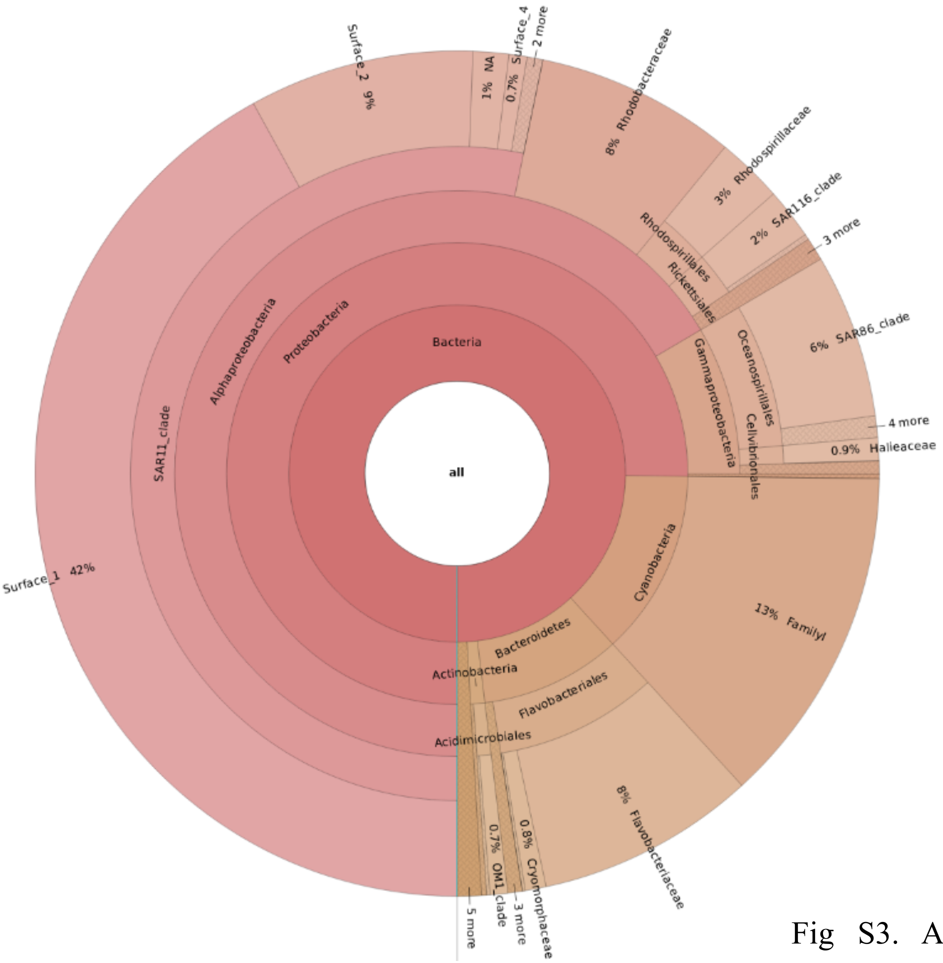

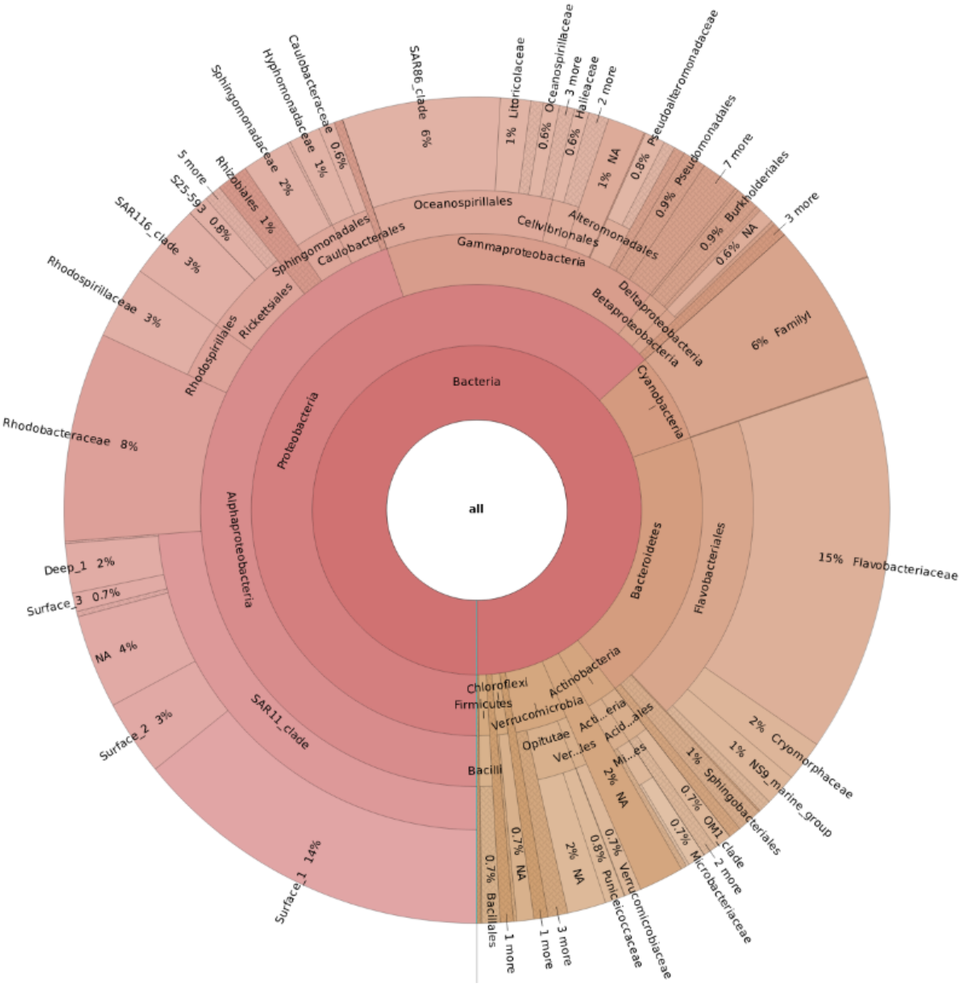

Fig S3. Abundant (top) and rare (bottom) communities deciphered by metabarcoding.

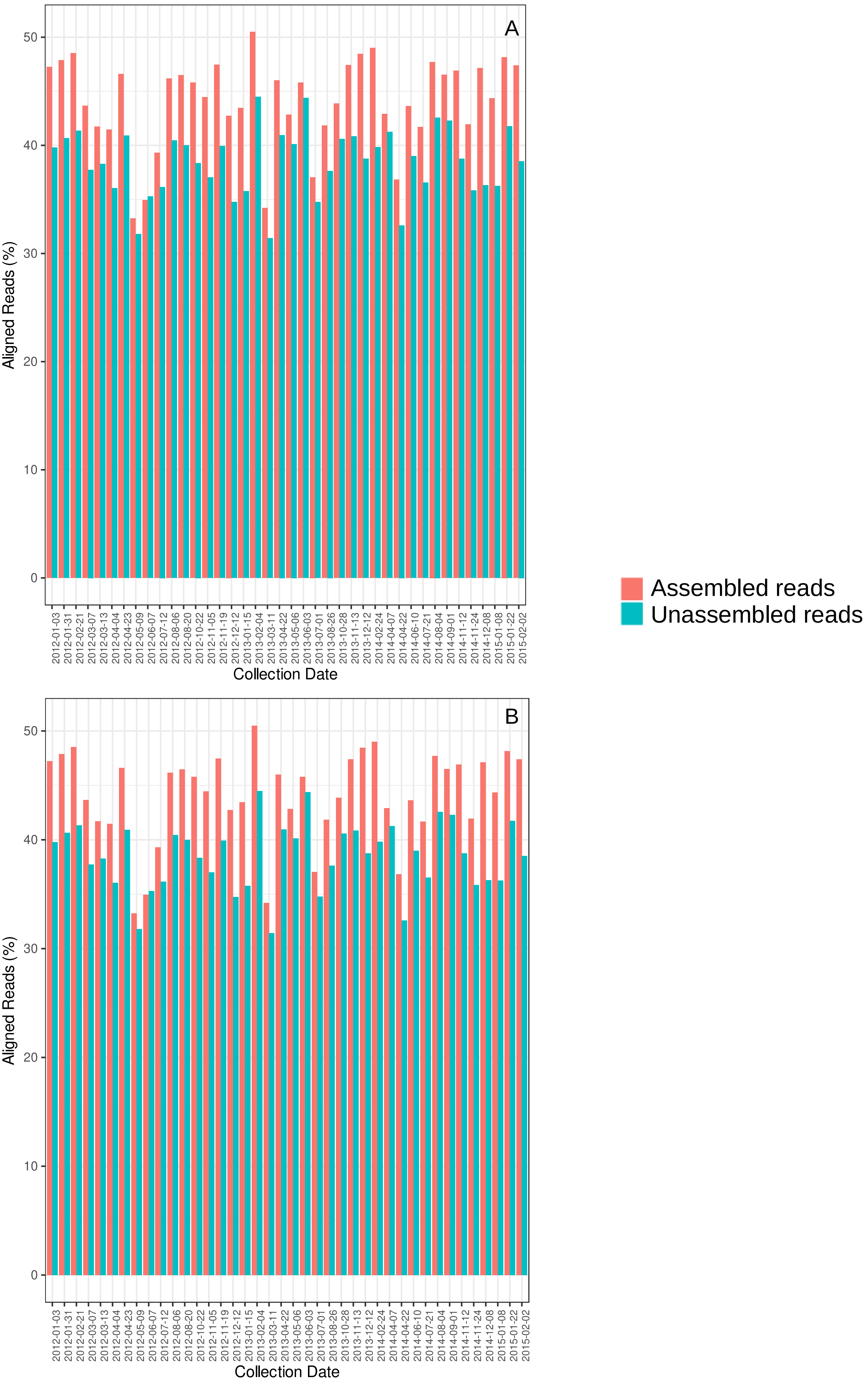

Fig S4. Distribution of the assembled and unassembled reads aligned to the UNIREF90 (A) and UNIREF100 (B) databases.

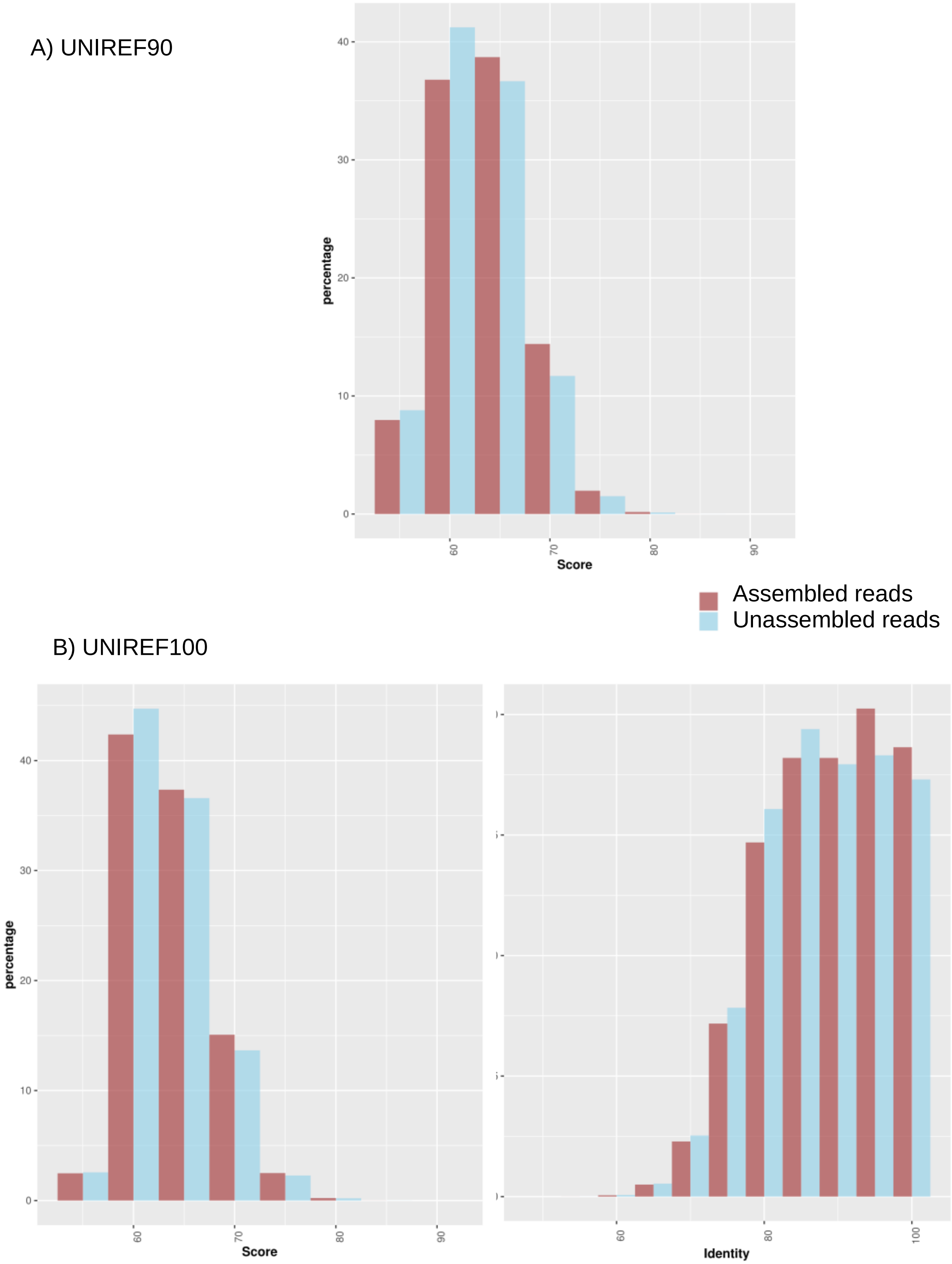

Fig S5. Distribution of the scores and identities of the assembled and unassembled reads aligned to the UNIREF90 (A) and UNIREF100 (B) databases.

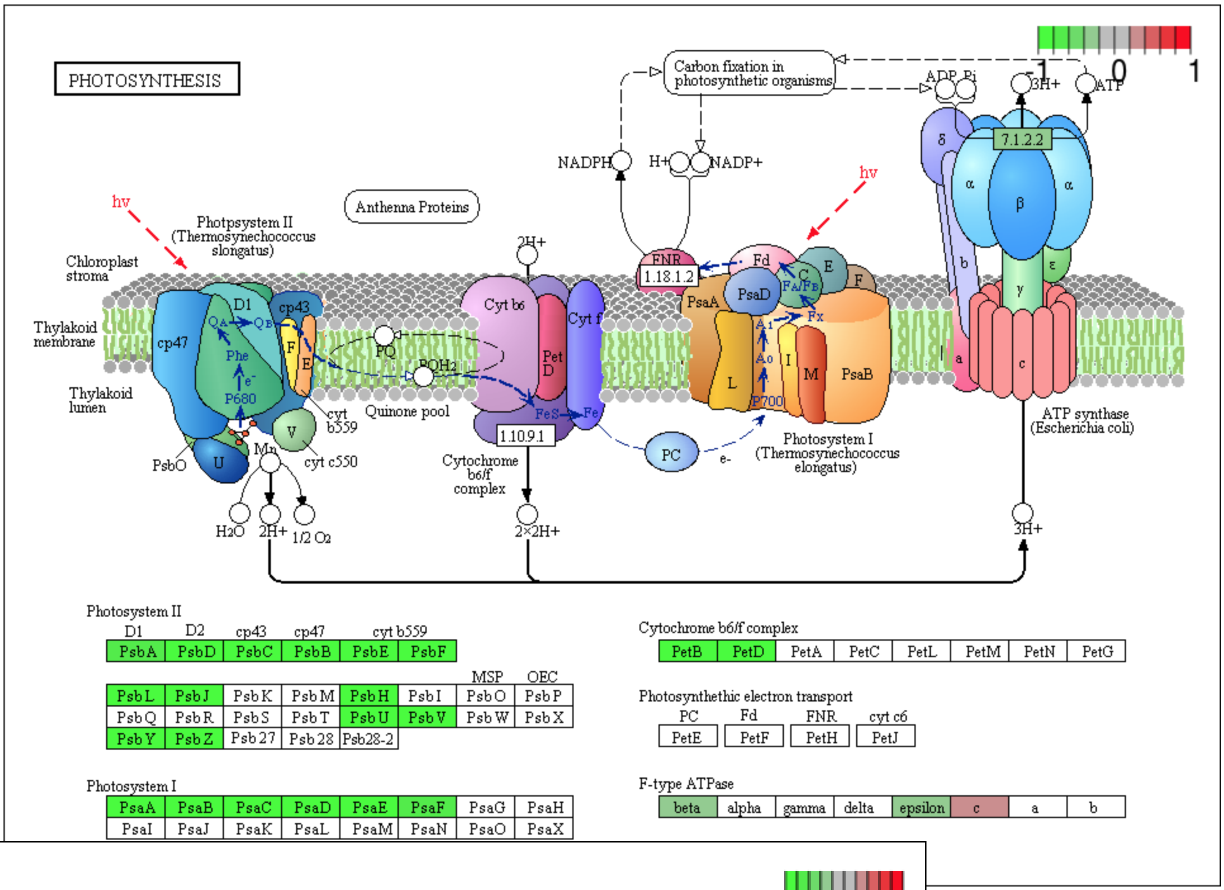

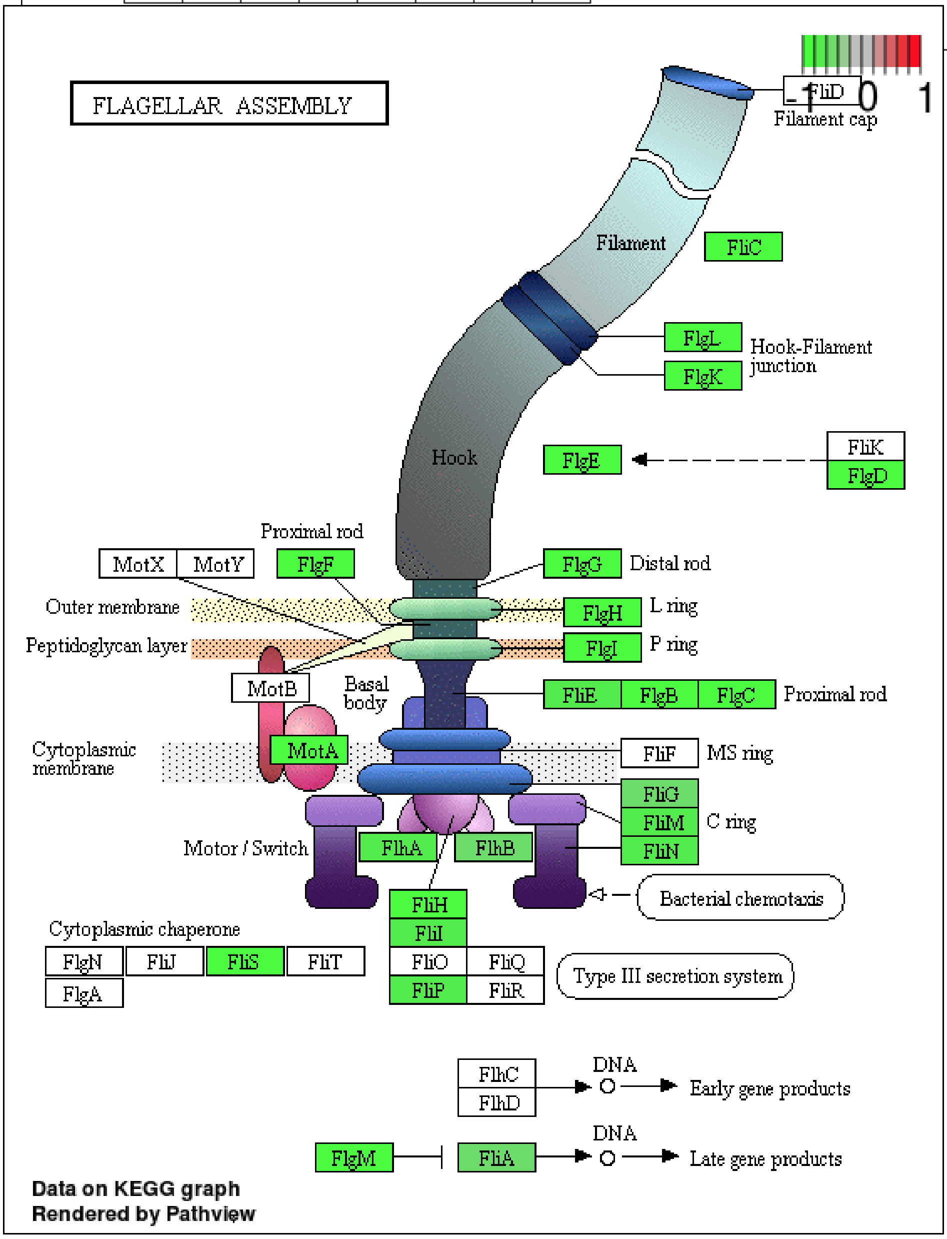

Fig S6. Photosynthesis and flagellar assembly pathways. Green rectangles corresponds to an enrichment in the assembled reads and red in the unassembled reads.

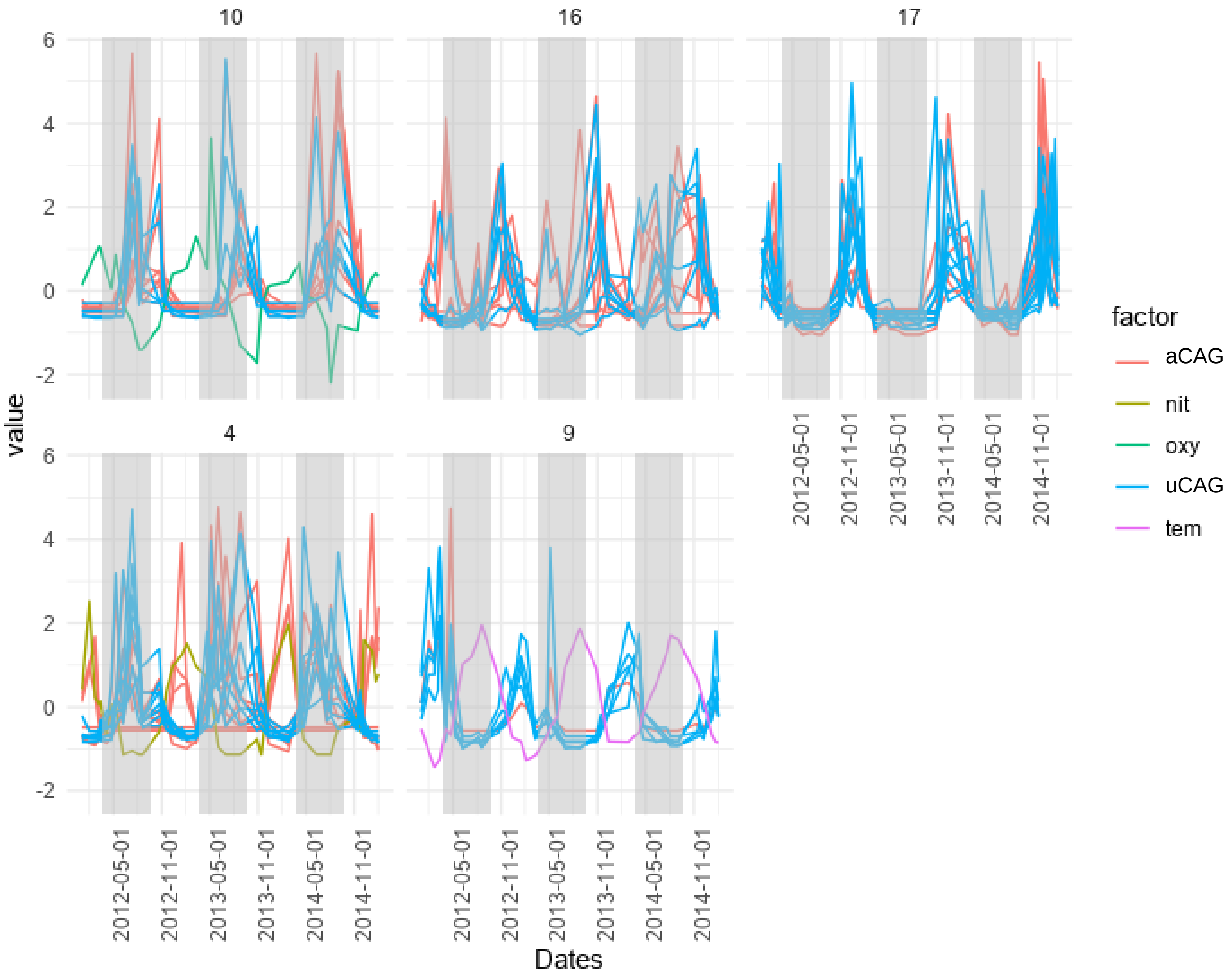

Pathways	KOs in pathways
**Cluster 16**	
ko00290 Valine, leucine and isoleucine biosynthesis	42%
ko00970 Aminoacyl-tRNA biosynthesis	32%
ko00780 Biotin metabolism	30%
**Cluster 17**	
ko00290 Valine, leucine and isoleucine biosynthesis	53%
ko00780 Biotin metabolism	39%
ko00970 Aminoacyl-tRNA biosynthesis	35%
ko00473 D-Alanine metabolism	33%
ko01230 Biosynthesis of amino acids	31%
ko00670 One carbon pool by folate	30%
ko00785 Lipoic acid metabolism	27%
ko00770 Pantothenate and CoA biosynthesis	26%
ko00195 Photosynthesis	25%
ko00860 Porphyrin and chlorophyll metabolism	25%

Fig S7 Temporal dynamics (z-scores) of the unassembled and assembled CAGs inside the main network clusters assessed by the Louvain methods. The clusters composed of less than 3 vertices are not represented. The grey rectangle represents spring and summer periods. The table displays the mains metabolic pathways in the clusters 16 and 17 (Any pathway with at least 25 % of the KOs were detected in the other clusters).

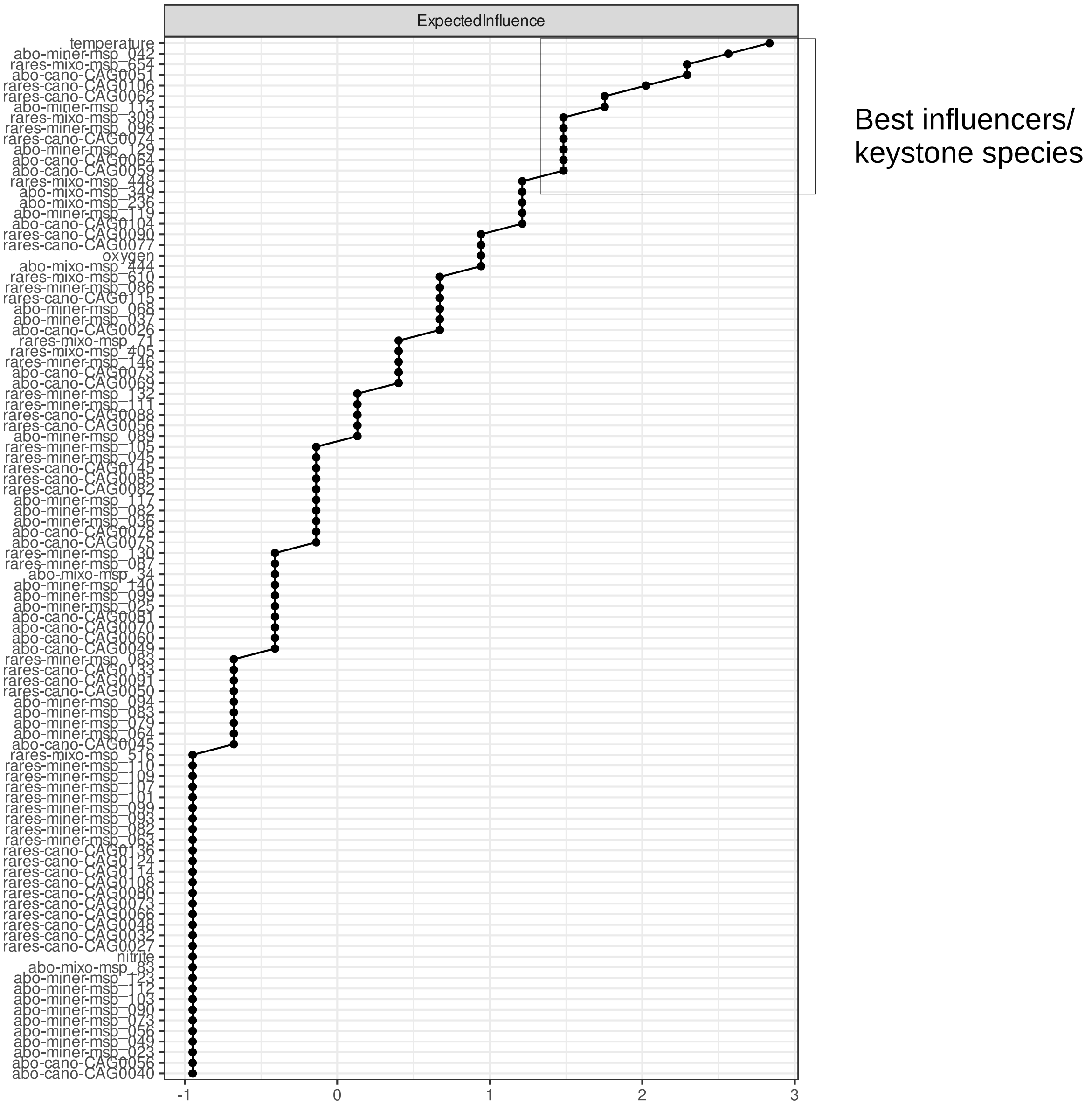

Fig S8. « ExpectedInfluence » parameter computed from the network with the package qgraph under R.
